# Scaling the *Drosophila* Wing: TOR-Dependent Target Gene Access by the Hippo Pathway Transducer Yorkie

**DOI:** 10.1371/journal.pbio.1002274

**Published:** 2015-10-16

**Authors:** Joseph Parker, Gary Struhl

**Affiliations:** 1 Department of Genetics and Development, Columbia University, New York, New York, United States of America; 2 Division of Biology, Imperial College London, London, United Kingdom; Rutgers, UNITED STATES

## Abstract

Organ growth is controlled by patterning signals that operate locally (e.g., Wingless/Ints [Wnts], Bone Morphogenetic Proteins [BMPs], and Hedgehogs [Hhs]) and scaled by nutrient-dependent signals that act systemically (e.g., Insulin-like peptides [ILPs] transduced by the Target of Rapamycin [TOR] pathway). How cells integrate these distinct inputs to generate organs of the appropriate size and shape is largely unknown. The transcriptional coactivator Yorkie (Yki, a YES-Associated Protein, or YAP) acts downstream of patterning morphogens and other tissue-intrinsic signals to promote organ growth. Yki activity is regulated primarily by the Warts/Hippo (Wts/Hpo) tumour suppressor pathway, which impedes nuclear access of Yki by a cytoplasmic tethering mechanism. Here, we show that the TOR pathway regulates Yki by a separate and novel mechanism in the *Drosophila* wing. Instead of controlling Yki nuclear access, TOR signaling governs Yki action after it reaches the nucleus by allowing it to gain access to its target genes. When TOR activity is inhibited, Yki accumulates in the nucleus but is sequestered from its normal growth-promoting target genes—a phenomenon we term “nuclear seclusion.” Hence, we posit that in addition to its well-known role in stimulating cellular metabolism in response to nutrients, TOR also promotes wing growth by liberating Yki from nuclear seclusion, a parallel pathway that we propose contributes to the scaling of wing size with nutrient availability.

## Abstract 2

From dwarves to giants, scaling is a universal property of animal organs, but its mechanistic basis is poorly understood. Here, the authors identify a molecular circuit underlying scaling of the *Drosophila* wing.

## Author Summary

What mechanisms control the sizes of animal organs? It is known that organ growth is the product of two systems: an intrinsic system that coordinates cell proliferation with the specification of cell fate (patterning), and an extrinsic system that synchronizes growth with nutrient levels. Developing organs integrate these two inputs to ensure that properly proportioned structures develop which are of the right scale to match overall body size. However, the mechanisms used to integrate these distinct growth control systems have remained largely mysterious. In this study, we have addressed how intrinsic and extrinsic systems combine to drive growth of the *Drosophila* wing. Focusing on the Target of Rapamycin (TOR) pathway—a major, nutrient-dependent regulator of organ growth—and Yorkie—the transcriptional activator downstream of the Hippo pathway and a key, organ-intrinsic growth regulator—we have identified a circuit in which TOR activity limits Yorkie’s capacity to promote wing growth, in part through a novel mode of transcription factor regulation that we term “nuclear seclusion.” We find that inhibiting TOR leads to the retention of Yorkie in the nucleus but diminishes its transcriptional activity by diverting it away from target genes. We posit that subjugating Yorkie in this way contributes to how fluctuations in TOR activity scale wing size according to nutrient levels.

## Introduction

A universal property of animal development is the capacity to scale body size and pattern in response to environmental conditions as well as during evolution [1]. For example, when starved during the larval growth phase, *Drosophila* adults emerge at around one quarter of their normal size but are correctly proportioned. Likewise, *Drosophila* species differ over ~5-fold in body size but are highly similar in shape. The overt likeness of primate skeletons (differing 18-fold in length), as well as those of frogs (30-fold) and fish (1,600-fold) provide more dramatic demonstrations of scaling across vast taxonomic groups, but despite its generality, the genetic control of organ scaling is poorly understood. Animals possess distinct systems for controlling growth locally and systemically: organ-intrinsic signaling mechanisms couple growth to patterning and morphogenesis, defining organ shape and dimension [2–4]; conversely, humoral signals, produced on feeding, act globally to control body size [5]. Yet, the existence of scaling implies the two systems cannot be independent. Cells in developing organs must integrate local and global information and proliferate accordingly, generating organs—and entire animals—that are functioning, proportional wholes [6–9].

**Fig 1:**
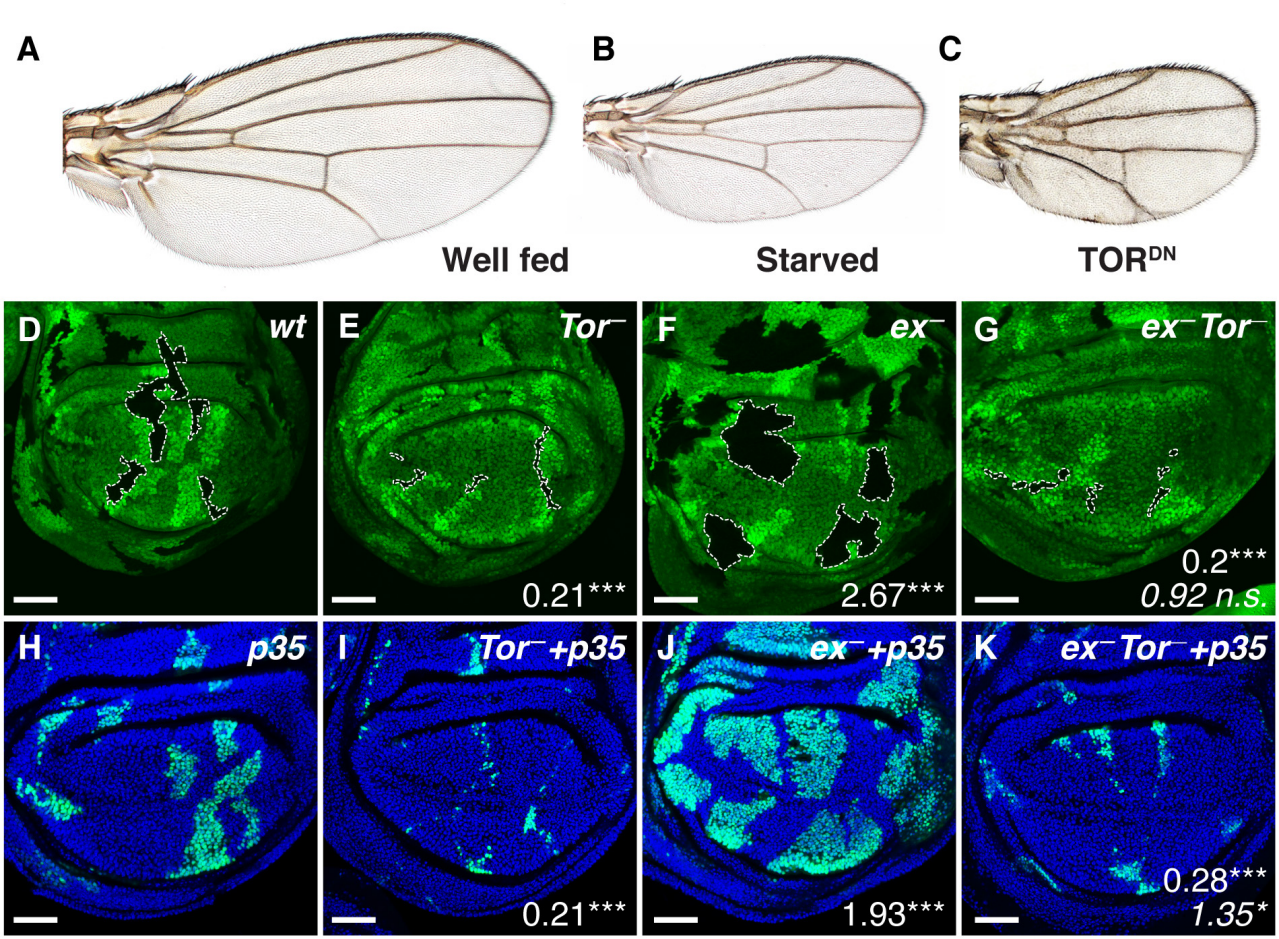
Effects of TOR inhibition on wing growth and Yki-driven cell proliferation. **A–C**: Adult *Drosophila* wings. (**A**) A wing from a well-fed *wild type* fly. (**B**) Scaled-down wing produced by raising larvae on nutrient-poor food. (**C**) Scaled-down wing caused by blocking TOR signaling specifically in wing cells with *nubbin*.*GAL4 UAS*.*Tor* ^*TED*^. (**D–G**) Wing discs from late third instar larvae-bearing clones of mutant tissue outlined with dashed lines, and marked negatively (“black”) by absence of the GFP marker (green): (**D**) *wild type* (control) (**E**) *Tor* ^*ΔP*^, (**F**) *ex* ^*e1*^, (**G**) *ex* ^*e1*^ *Tor* ^*ΔP*^. Mutant clones were induced at the end of the first instar, 48±2 hr after egg laying and are associated with sibling “twin-spot” clones marked by two copies of the GFP marker (bright green) that serve as an internal control for the growth of *w*.*t*. tissue. Numbers denote mean clone size ratio compared to *wt*, and asterisks denote significances from *t* tests (* = *p* < 0.05, ** = *p* < 0.01, *** = *p* < 0.001, *n*. *s*. = not significant). In (**G**), the bottom italicised value is a comparison with the *Tor* ^*ΔP*^ genotype. Number of clones measured (*n*) = 32 (*wt*), 38 (*ex*), 51 (*Tor*), 36 (*ex Tor*). (**H–K**) Clones of the same genotypes as in (**D**–**G**) that coexpress p35 with GFP-NLS (generated using the MARCM technique [36]. Clones are positively labelled by GFP-NLS, and nuclei are counterstained with Hoechst (blue). (*n*) = 92 (*wt+p35*), 90 (*ex+p35*), 97 (*Tor+p35*), 79 (*Ex Tor+p35*). Numbers signify mean clone size ratio compared to *wt+p35*, and bottom italicised value in (**K**) with *Tor+p35*.

The *Drosophila* wing is a classical paradigm of organ growth [2,3]. Here, as in other animals, nutrients influence growth via Target of Rapamycin (TOR) signaling [5,10]. During larval life, this pathway is activated in wing cells by haemolymph signals produced in response to feeding, including Insulin-like peptides (ILPs) that act via the Insulin Receptor (InR)/PI3-Kinase/Akt pathway, as well as sugars and amino acids. These inputs converge to regulate TOR—an intracellular kinase with diverse roles in metabolism [5,10]. Starvation reduces TOR activity and scales wing size (and entire body size) downwards (Fig 1A and 1B), an effect mimicked by genetically inhibiting TOR (Fig 1C). Yet, wing growth is simultaneously governed by intrinsic signaling systems (e.g., Wnt, BMP, and Hh morphogens) that control wing size, shape, and pattern [2,3,6,8]. Many of these organ-intrinsic systems exert their effects at least in part via regulation of the Warts (Wts)/Hippo (Hpo) pathway [11–13]—a network of proteins that inhibit a growth-promoting transcriptional coactivator, Yorkie (Yki; orthologous to vertebrate YES-Associated Protein [YAP]) [14]. Hpo and Wts are kinases that act in sequence, Hpo phosphorylating Wts and Wts phosphorylating Yki to sequester Yki cytoplasmically. Inhibition of either kinase promotes growth by allowing Yki to evade cytosolic sequestration and gain access to the nucleus [15–17]. Nuclear Yki binds transcription factors including Scalloped (Sd, a TEAD [transcriptional enhancer activator domain] protein) [18,19] to up-regulate expression of genes that promote cell growth and proliferation. Morphogens [20,21], the protocadherins Fat and Dachsous [22–25], the Crumbs/Lgl epithelial polarity proteins [26–29], and mechanical strain [30] all modulate either or both Wts and Hpo, exerting effects on wing growth via Yki. But for the wing to scale, Yki activity, or the growth that Yki stimulates, must be contingent on TOR activity. Despite previous attempts to assess the links between Wts/Hpo and InR/TOR signaling [15,31–35], the logic by which cells in growing organs integrate these inputs to achieve organ scaling remains unclear.

Here, we report a novel molecular mechanism by which Yki can integrate the control of *Drosophila* wing growth by the Wts/Hpo and TOR pathways. As part of this mechanism, we present evidence for what is, to our knowledge, a previously unknown mode of transcriptional regulation, which we term “nuclear seclusion”, whereby a transcription factor (Yki) is retained in the nucleus but sequestered from target loci and hence rendered unable to induce their expression. In contrast to the Wts/Hpo pathway, which controls Yki activity by governing its access to the nucleus, our findings indicate that the TOR pathway activates Yki after it gains entry to the nucleus, by allowing it to escape nuclear seclusion and gain access to its growth-promoting target genes. We posit that this novel mechanism allows Yki to integrate local patterning inputs mediated by the Wts/Hpo pathway with systemic, humoral inputs mediated by TOR, comprising part of the system that scales wing growth in response to nutrition.

## Results

### TOR Activity Is Required for Yki-Dependent Wing Growth

To determine the relationship between TOR and Yki in the developing *Drosophila* wing, we first asked whether the capacity of Yki to promote growth depends on TOR activity. To do so, we generated clones of cells mutant for Wts/Hpo pathway components that normally hold Yki activity in check and tested if the resulting increases in tissue mass and cell number depend on TOR activity. All such experiments that we performed indicate an obligate role of TOR activity for Yki-dependent wing growth. Specifically, clones mutant for the FERM (4.1 Ezrin Radixin Moesin) domain protein Expanded (Ex), which is required for normal Hpo activity, grow far larger than control clones (Fig 1F; S1 Data [37]). However, when such clones were also mutant for TOR (*ex* ^*—*^ *Tor* ^*—*^clones), they were tiny (Fig 1G; S1 Data) and not significantly larger than *Tor* ^—^clones (Fig 1D and 1E; S1 Data). The impaired growth of both *Tor* ^*—*^and *ex* ^*—*^ *Tor* ^*—*^clones is not due to apoptosis, since it is not detectably rescued by expressing the apoptosis inhibitor p35 in the mutant cells (Fig 1H–1K; S1 Data). Equivalent experiments, activating Yki by removing Wts and inhibiting TOR by removing the TOR-activating GTPase Rheb, gave similar results (S1A–S1H Fig). Likewise, overexpressing Yki throughout the wing primordium leads to excessive growth; however, co-overexpression of ΔP60, an inhibitor of InR signaling upstream of TOR [38], blocks this growth, yielding wings similar in size to those in which only ΔP60, alone, was overexpressed (S1I–S1O Fig and discussion in S1 Fig legend; S1 Data). Equivalent results were obtained in the developing head primordium: mutation of *wts* or *ex* leads to dramatic overgrowth of head tissue, which is suppressed when the head is also mutant for *Tor* or the InR pathway kinase Akt (S1P–S1X Fig). Thus, reducing the level of InR and TOR signaling, which is normally set by nutrient status, restricts Yki’s capacity to promote growth—suggesting a relationship that could contribute to scaling wing size with nutrient levels.

### Yki Does Not Up-Regulate InR/TOR Pathway Activity to Promote Growth

**Fig 2:**
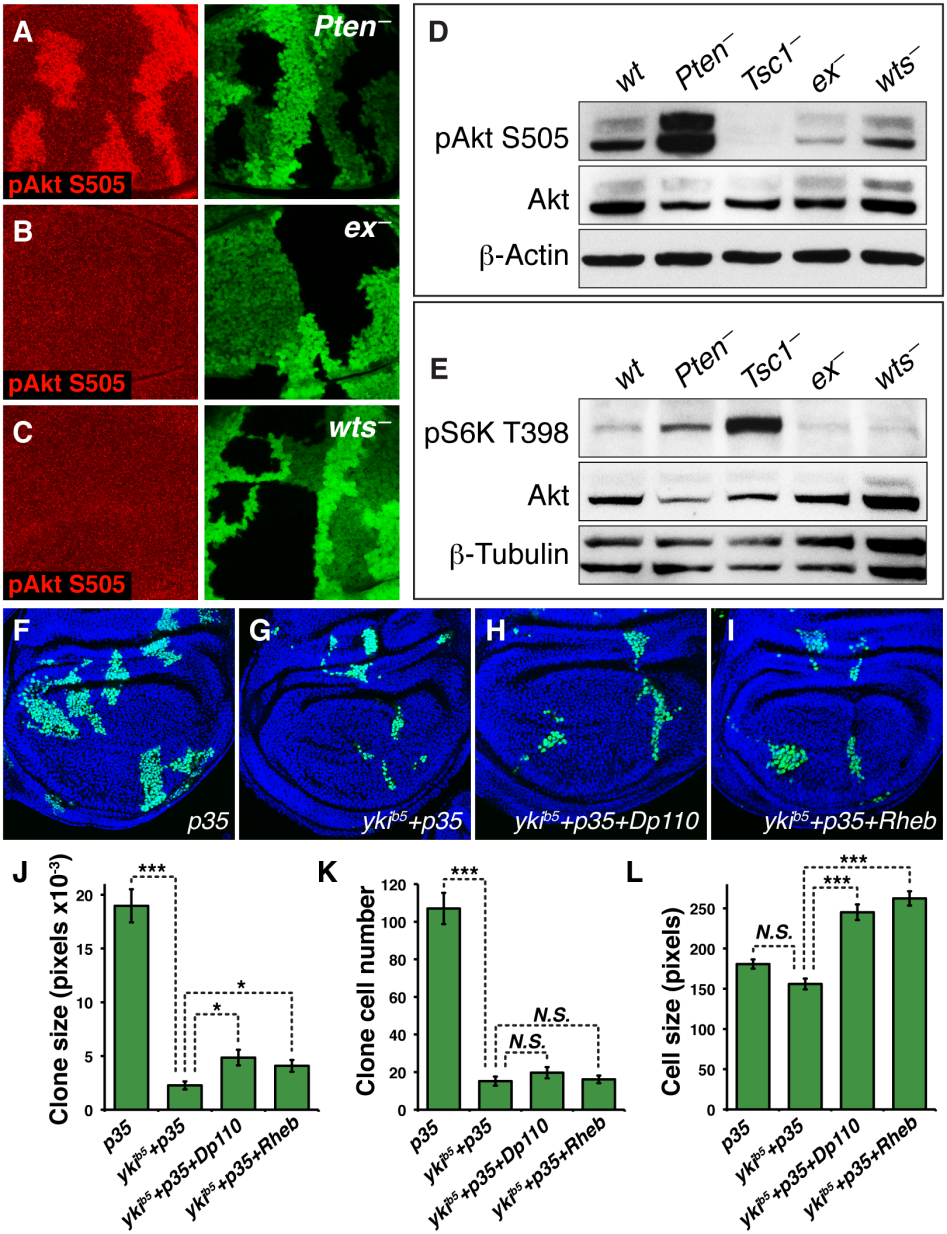
Yki activity does not promote growth by up-regulating InR/TOR signaling. **(A–C)** Wing discs labelled for phospho-Akt S505, an indicator of InR pathway activity, bearing mutant clones marked black by absence of GFP (green); *w*.*t*. twin spots are marked by bright green. (**A**) *pten* ^*1*^ (positive control), (**B**) *ex* ^*e1*^, and (**C**) *wts* ^*X1*^ (experimentals, having elevated Yki activity owing to reduced or absent phosphorylation by Wts). Phospho-Akt S505 is not increased in (**B**) and (**C**), in contrast to (**A**). **(D)** Western blot of protein extracts derived from late third instar wing discs of the genotypes shown, labelled for phospho-Akt S505 (Total Akt and β-actin were used as loading controls; *pten* ^*1*^/*pten* ^*dj189*^ was used as a positive control; the reduction in S505 staining in the *Tsc1* ^*Q87X*^/*Tsc1* ^*PA23*^ lane is due to feedback of TOR activation onto Akt phosphorylation [39]). In contrast to reduced Pten, loss of either Ex or Wts does not cause an increase in pAkt S505. **(E)** Blot of same genotypes as in (**D**), labelled for phospho-S6 Kinase T398, an indicator of TOR pathway activity (β-Tubulin was used as a loading control, and runs as two species). As observed for phospho-Akt S505, Phospho-S6 Kinase T398 levels are elevated by loss of Pten and Tsc activity, but not by loss of either Ex or Wts activity. (**F–I**): Wing discs from late third instar larvae-bearing MARCM clones expressing *UAS*.*p35* (labelled positively with GFP-NLS, green; nuclei are counterstained with Hoechst, blue). The genotypes of clones are (**F**) *UAS*.*p35*, (**G**) *yki* ^*b5*^+*UAS*.*p35*, (**H**) *yki* ^*b5*^+ *UAS*.*p35+UAS*.*Dp110*, and (**I**) *yki* ^*b5*^ *+ UAS*.*p35+UAS*.*Rheb*. **J–L**: Quantification of clones sizes, cell numbers, and cell sizes from genotypes in F–I. Error bars are Standard Error of the Mean and asterisks denote significances from *t* tests (* = *p* < 0.05, ** = *p* < 0.01, *** = *p* <0.001, *n*. *s*. = not significant). *n* = 42 (*p35*), 49 (*yki* ^*b5*^ *+p35*), 76 (*yki* ^*b5*^ *+p35+Dp110*), 76 (*yki* ^*b5*^ *+p35+Rheb*). Expression of either *UAS*.*Dp110* or *UAS*.*Rheb* results in an increase in the size of *yki* mutant clones caused by an increase in cell size but not cell number.

Why is Yki-driven growth limited by the level of TOR activity? TOR might be required upstream to facilitate Yki activity, or in parallel to trigger other, independent growth-related processes. Alternatively, TOR might be required downstream, with Yki promoting growth at least in part by elevating InR/TOR pathway activity. To test this latter possibility, we assayed whether conditions that abnormally increase Yki activity (loss of either Ex or Wts) have a corresponding effect on the levels of phospho-Akt (pAkt-S505), which are normally elevated by enhanced InR signaling (e.g., by removal of the InR pathway inhibitor PTEN; Fig 2A). We find that pAkt-S505 levels are normal in *ex* ^—^or *wts* ^—^clones (Fig 2B and 2C), as well as in protein extracts from the overgrown wing discs of homozygous *ex* ^—^or *wts* ^—^larvae (Fig 2D). Likewise, the levels of phosphorylated S6-Kinase (pS6K-T398), a readout of TOR activity, were not affected in *ex* ^—^or *wts* ^—^discs (Fig 2E). We therefore infer that Wts/Hpo regulated Yki activity does not stimulate either InR or TOR pathway activity in the wing, consistent with results from *Drosophila* cell culture [15]. This finding argues against a downstream role of InR/TOR signaling in mediating Yki-driven growth.

Further evidence that TOR activation is not dependent on Yki comes from the observation that ectopically activating the InR/TOR pathway via upstream components can suffice to increase the growth of *yki* ^—^wing clones (rescued with p35; Fig 2F, 2G and 2J; S1 Data). Specifically, expressing Rheb (to activate TOR) resulted in a 22% increase in clone size (Fig 2I and 2J; S1 Data) and expressing Dp110 (to activate InR) resulting in a 26% increase (Fig 2H and 2J; S1 Data). In both cases, clonal expansion resulted solely from increases in cell size (Fig 2L; S1 Data), without effects on cell number (Fig 2K; S1 Data). Hence, TOR can be activated and promote detectable cell growth, albeit not cell proliferation, in the absence of Yki. Notably, control clones expressing Dp110 or Rheb with p35 that are wild type for Yki strongly overgrew (S2A–S2D Fig; S1 Data), and this was in part due to both their increased cell numbers (S2E Fig; S1 Data), as well as strongly increased cell size (S2F Fig; S1 Data). We infer that although InR/TOR pathway activity appears largely independent of Yki activity, ectopic InR/TOR activation nevertheless requires Yki function as a prerequisite in order to drive cell proliferation.

### TOR Inhibition Enhances Yki Nuclear Accumulation despite Inhibiting Yki-Dependent Growth

**Fig 3:**
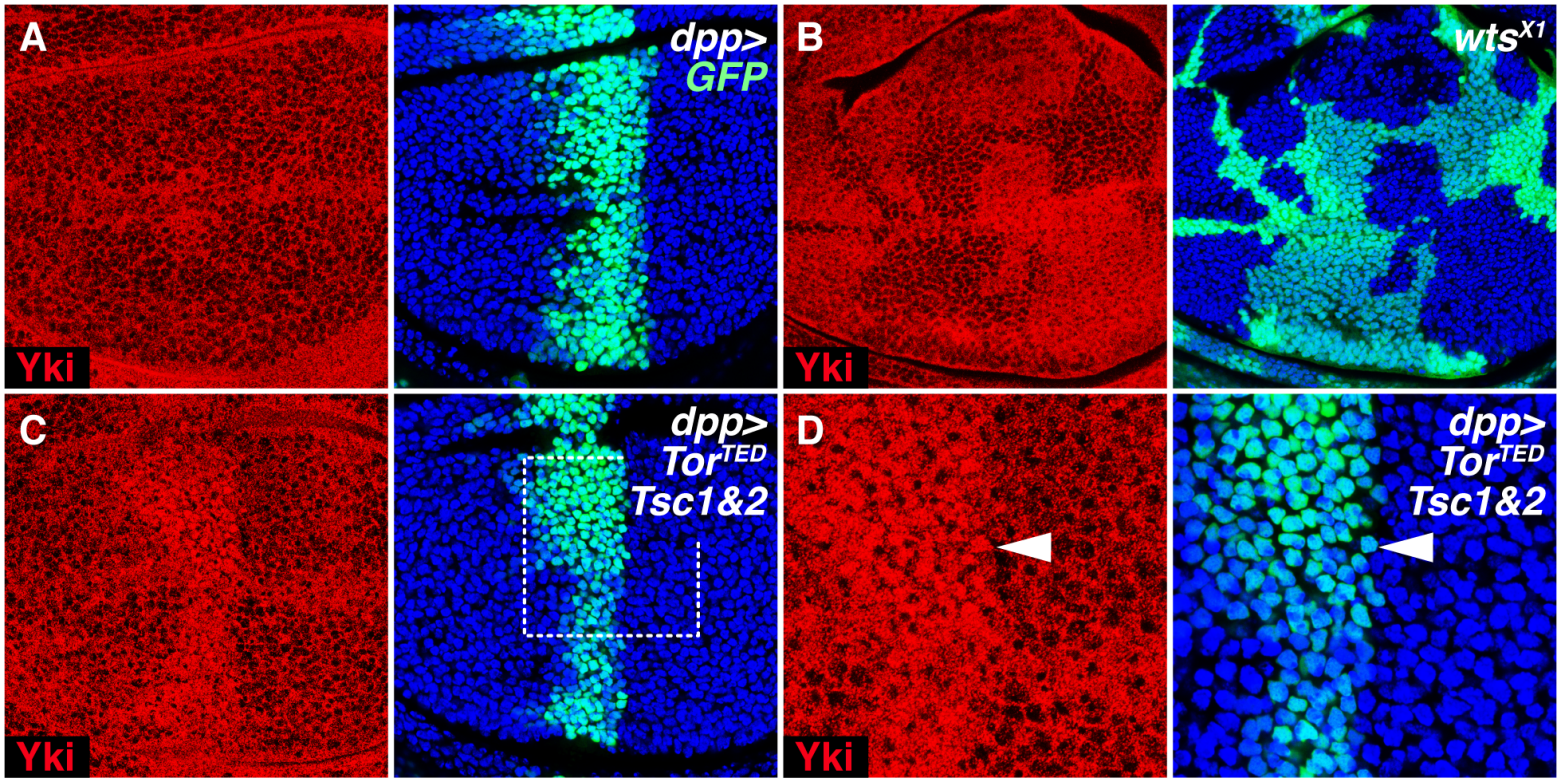
TOR inhibition increases Yki nuclear accumulation. **(A,C,D)** Wing discs expressing GFP-NLS in a stripe of cells under *dpp*.*Gal4* control that either do (**C,D**, experimental), or do not (**A**, negative control), coexpress *Tor* ^*TED*^, *TSC1*, and *TSC2*. (**B**) Wing disc with *wts* ^*X1*^ clones (positive control), marked black by the absence of GFP. Discs are imaged at the level of nuclei and labelled for Yki (red), GFP (green), and DNA (Hoechst, blue). Coexpression of *Tor* ^*TED*^, *TSC1*, and *TSC2* (**C,D**), like the loss of Wts (**B**), causes enhanced nuclear accumulation of Yki, apparent at this plane of focus as increased signal at low magnification (**B,C**) and by coincidence of the Yki and GFP-NLS signals at high magnification (**D**, e.g., arrowhead).

If Yki depends on, but does not activate, the InR/TOR pathway to promote growth, an alternative explanation is that the InR/TOR pathway acts in parallel to or upstream of Yki. Yki activity depends in large part on phosphorylation by Wts, which causes Yki to be sequestered in the cytosol, reducing its access to the nucleus [15–17]. Yki normally appears predominantly cytoplasmic in the developing wing (Fig 3A), consistent with its essential, but tonic, activity in sustaining wing growth. However, upon Wts inactivation, Yki accumulates in the nucleus and inappropriately up-regulates transcription of its growth-promoting target genes, causing pronounced tissue hyperplasia [15–17] (Fig 3B). Hence, inhibiting TOR activity might countermand the overgrowth phenotype caused by loss of Wts activity by compromising nuclear access of unphosphorylated Yki. In preparing to test this possibility, we were surprised to discover that reducing TOR activity has the opposite effect on Yki localization: instead of restricting nuclear access, it causes a dramatic increase in nuclear Yki, even as it blocks Yki-dependent growth.

In our initial experiments, we strongly inhibited endogenous TOR activity in the developing wing, using *dpp*.*Gal4* to drive a stripe of expression of *UAS* transgenes encoding three proteins that have been widely employed to study the consequences of reducing TOR activity: a dominant negative form of TOR (TOR^TED^) and the TOR corepressors TSC1 and TSC2 [32,40–46]. Co-overexpression of all three proteins causes strong nuclear Yki accumulation in the stripe (Fig 3C and 3D). Expressing either TSC1+TSC2, or TOR^TED^, alone, in the stripe or in other patterns within the wing, also caused enhanced Yki nuclear localization (S3A–S3C Fig), albeit less strongly, suggesting that the level of nuclear accumulation depends on the strength of TOR inhibition, and furthermore is not region-specific. Note that we prefer the use of transgenes to inhibit TOR signaling in large sectors of tissue, rather than examining tiny *Tor* ^—^clones, where interpretation is compromised by protein perdurance. We also observed enhanced Yki nuclear accumulation when InR signaling was inhibited, upstream of TOR (S3D–S3F Fig). Conversely, removal of two downstream metabolic targets of TOR, dS6K, and 4EBP/Thor [10] did not affect Yki nuclear accumulation (S3G–S3J Fig), indicating that the control of Yki localization by TOR involves a mechanism that is not mediated by these metabolic regulators. Thus, reduced InR/TOR activity results in enhanced nuclear accumulation of Yki—a result that is counterintuitive, because nuclear Yki is usually associated with increased, rather than reduced growth [15–17].

### TOR Inhibition Reduces Yki Target Gene Transcription

**Fig 4:**
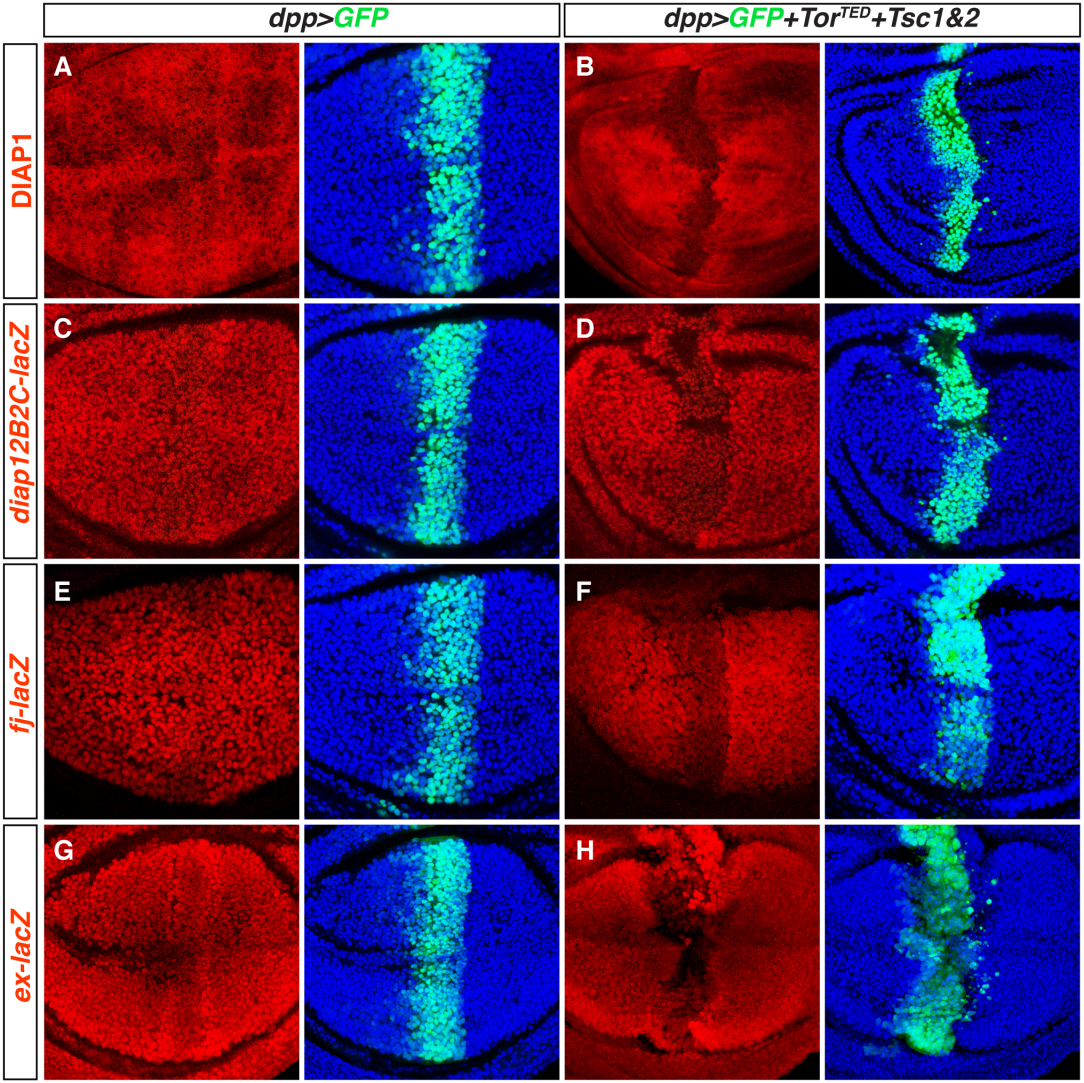
TOR inhibition reduces Yki target gene expression. Wing discs expressing GFP-NLS (green) under *dpp*.*Gal4* control with (**B, D, F, H**), or without (**A, C, E, G**), coexpressing the TOR inhibitors *Tor* ^*TED*^, *TSC1* and *TSC2*, as in Fig 3. TOR inhibition reduces the Yki targets DIAP1 protein (**B**), *diap1* ^*2B2C*^ *-lacZ* (**D**) *fj-lacZ* (**F**) and *ex-lacZ* (**H**; kept for 8 hr at 29°C before dissection, to increase the level of TOR inhibition), all labelled in red (nuclei counterstained with Hoechst, blue).

Typically, nuclear Yki up-regulates growth-promoting target genes, one of the best characterized of these being *diap1*, the gene encoding the *Drosophila* Inhibitor of Apoptosis protein [18,19]. However, the dramatic increase in nuclear Yki accumulation caused by TOR inhibition (Fig 3C and 3D) is associated with a strong reduction in DIAP protein levels (Fig 4A and 4B), consistent with reduced *diap1* gene transcription. *Diap1* transcription is normally driven by a Yki-Sd complex that binds ~4 kb downstream of the transcription start site of the predominant *diap1*-RA transcript [19]. A minimal reporter construct, *2B2C-lacZ*, containing ~300 bp of this enhancer region, is up-regulated by Yki overactivation [19]. Conversely, RNAi knockdown of Yki results in reduced *2B2C-lacZ* expression (S4A and S4A’ Fig). Like Yki RNAi knockdown, TOR inhibition caused a marked decrease in *2B2C-lacZ* expression (Fig 4C and 4D), indicating a failure of the Yki/Sd-dependent *2B2C* enhancer to maintain normal target gene expression.

TOR inhibition similarly compromises the transcription of other Yki target genes. *four-jointed* (*fj*) is the second target gene that we have confirmed is reduced by Yki knockdown (S4B Fig). Like Yki knockdown, TOR inhibition strongly reduced *fj-lacZ* expression (Fig 4E and 4F). A third reporter, *ex* ^*e1*^ *-lacZ*, was unresponsive to TOR inhibition at 25°C, but raising the temperature to 29°C for several hours prior to dissection (to increase GAL4-dependent TOR^TED^, TSC1, and TSC2 overexpression) sufficed to repress this reporter (Fig 4G and 4H). Further, in animals homozygous for the *ex* ^*e1*^ *-lacZ* reporter allele, *ex* ^*e1*^ *-lacZ* expression was clearly reduced by TOR inhibition at 25°C (S4C and S4D Fig). We note that such *ex* ^*e1*^ *-lacZ*/*ex* ^*e1*^ *-lacZ* discs have reduced *ex* gene function and hence abnormally elevated levels of nuclear Yki; nevertheless, a sharp increase in nuclear Yki can still be detected in the TOR-inhibited stripe, coincident with reduced transcription of the *ex* ^*e1*^ *-lacZ* gene (S4E–S4G Fig). Finally, the effect of TOR inhibition appears specific to Yki target genes, since other proteins expressed in the developing wing, including Vestigial (S4I Fig), Armadillo (S4J Fig), Distal-less (S4J’ Fig), a *Distal-less-lacZ* reporter (S4K Fig), and indeed Yki itself (Fig 3C and 3D), were not reduced by strong TOR inhibition.

### Inhibiting TOR Impedes Target Gene Access by Yki

The unexpected (and counterintuitive) effects of TOR inhibition on Yki nuclear accumulation and target gene expression are reminiscent of another scenario in which enhanced nuclear access of Yki is associated with the loss of target gene expression. In this case, Yki can be forced to accumulate in the nucleus by overexpression of Sd, the site-specific DNA binding factor that normally mediates target gene activation by Yki in the wing blade (S6A Fig) [18,19]. Under this condition, the abnormally high levels of Sd sequester the limited levels of Yki available, exerting a classic dominant negative “squelching” effect [47] that titrates Yki away from Sd bound to target gene enhancers. As in the case of TOR inhibition, the level of nuclear Yki increases whilst target gene expression decreases (S6B and S6C Fig). This resemblance suggests that TOR inhibition may exert its effects on Yki target gene expression via a similar mechanism: by directing Yki to the nucleus whilst reducing its access to the relevant target gene enhancers.

**Fig 5:**
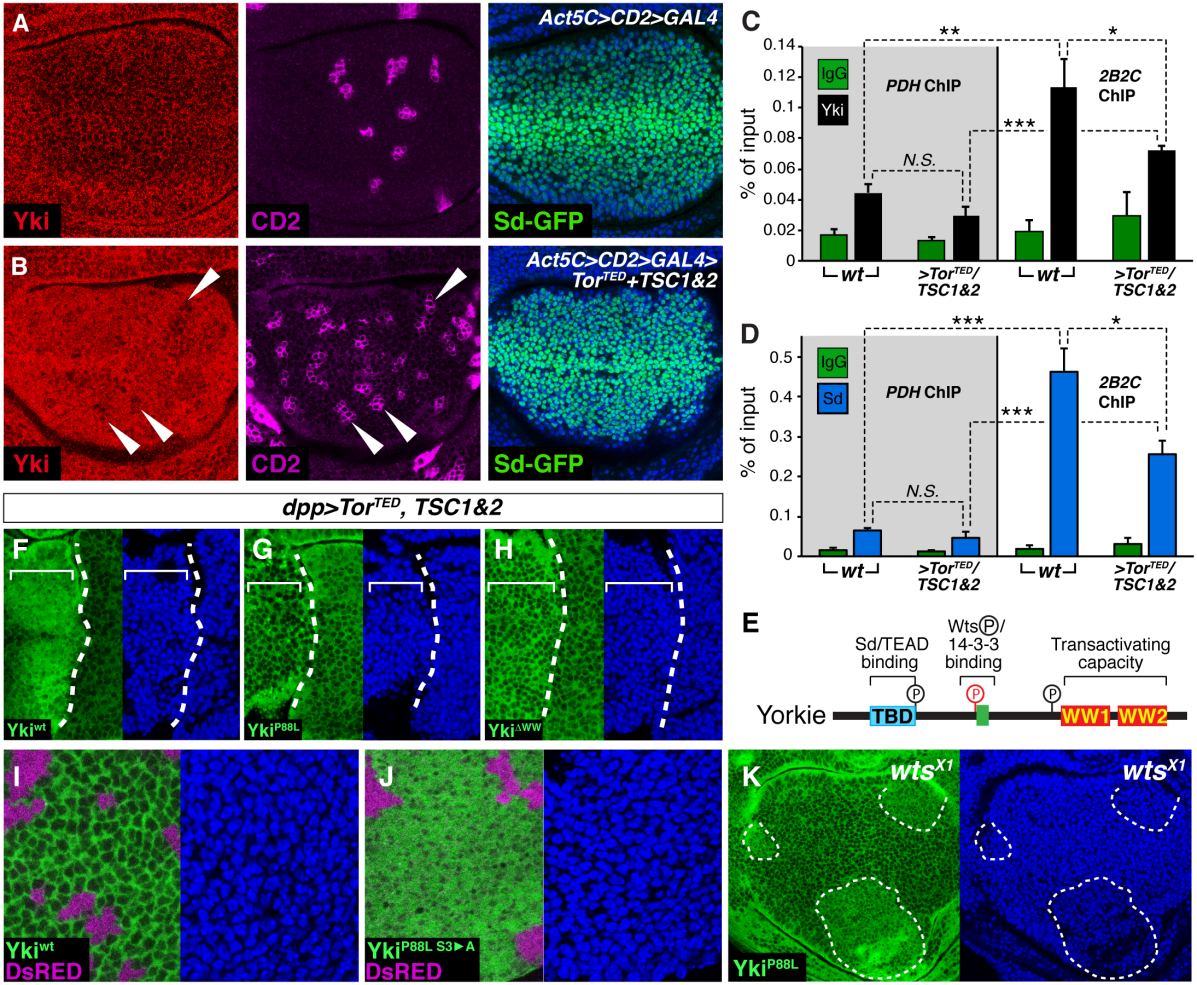
TOR inhibition results in Yki nuclear seclusion and depends on the conserved Sd binding and WW domains but not the Wts phosphorylation sites of Yki. **(A, B)** Confocal sections of *Act5C>CD2>GAL4* wing discs that either do (**B**, experimental) or do not (**A**, control) coexpress *Tor* ^*TED*^, *TSC1*, and *TSC2* in most cells 8–10 hr after heat shock to excise the *>CD2>* stop cassette. Yki (red), CD2 (magenta), Sd^GFP^ (from a GFP knockin allele of *sd*; green) and DNA (blue) are shown at the level of nuclei. Control discs (expressing only GFP-NLS) have no effect on Yki localistion (**A**) in contrast to experimental discs coexpressing TOR^TED^, TSC1, and TSC2, which show enhanced nuclear accumulation of Yki as indicated by increased signal at low magnification (**B**; arrowheads show clusters of *Act5C>CD2>GAL4* cells in which the *>CD2>* stop cassette was not excised, which coincide with reduced Yki nuclear staining). (**C, D**) ChIP of Yki (**C**) or Sd^GFP^ (**D**; using the *sd* ^*GFP*^ allele and anti-GFP antibody) with mock IP (IgG) at the *2B2C diap1* enhancer and a control locus (PDH: *pyruvate dehydrogenase*) in control and experimental discs, as in (**A,B**). Error bars are Standard Error of the Mean, and asterisks denote significances from *t* tests (* = *p* < 0.05, ** = *p* < 0.01, *** = *p* < 0.001, *n*. *s*. = not significant). *n* = 4 independent experimental replicates. Yki and Sd^GFP^ are strongly enriched at *2B2C* in control (*w*.*t*.) discs compared to PDH controls. Enrichment of both proteins is reduced in experimental (>*TOR* ^*TED*^ */TSC1&TSC2*) discs; (**E**) Conserved functional domains of Yki. (**F–H**) Wing discs uniformly expressing *w*.*t*. (**F**), P88L (**G**), or WW domain mutant (W292A P295A W361A P364A) (**H**) forms of GFP tagged Yki that also express TOR^TED^, TSC1, and TSC2 in a stripe under *dpp*.*Gal4* control (as in Figs 3 and 4; the dashed white lines indicate the A/P compartment boundary, which abuts the right edge of the *dpp*.*Gal4* expressing stripe). The discs are imaged at the level of nuclei and show nuclear accumulation of the *w*.*t*. (**F**) but neither the P88L (**G**) or WW (**H**) mutant, forms of Yki, as indicated by signal intensity. (**I, J**) Confocal sections of wing discs taken at the level of nuclei to the assess the nuclear accumulation of wild-type Yki-GFP (**I**), as well as a mutant form of Yki-GFP (**J**), that carries both the P88L substitution (which blocks nuclear accumulation in response to TOR inhibition, **G**) as well as the S111A, S168A, and S250A (S3->A) substitutions (which obviate phosphorylation of Yki by Wts and cause otherwise wild-type Yki to accumulate in the nucleus). Both proteins are expressed in clones under the direct control of the *Tubα1* promoter following Flp-out cassette excision of a *Tubα1>DsRed>yki-GFP* transgene: the clones are marked black by the absence of DsRed expression (magenta). Wild-type Yki-GFP (**I**) appears predominantly cytosolic, whereas Yki^P88L S3->A^-GFP (**J**) appears much more nuclear, as indicated by the difference in staining patterns imaged at the nuclear plane—largely absent in nuclei for (**I**) and relatively uniform for (**J**): hence, the P88L mutation, which blocks Yki nuclear accumulation in response to TOR inhibition does not preclude nuclear accumulation in the absence of phosphorylation by Wts. (**K**) Yki^P88L^-GFP expressing wing disc carrying clones of *wts* ^—^clones (outlined with dashed white lines). Nuclear accumulation of the Yki^P88L^-GFP protein is elevated in the absence of phosphorylation by Wts, as indicated by increased GFP signal intensity imaged at the nuclear plane, corroborating the results in (**I,J**).

To test this possibility directly, we focused on *diap1*, the locus where the general mechanism of transcriptional control by Yki is best characterized, with a defined enhancer element, *2B2C*, that is controlled exclusively by Yki in complex with Sd (henceforth Yki-Sd) [14,18,19,48]. We prepared chromatin from control and TOR-inhibited wing discs (the latter coexpressing TOR^TED^+TSC1+TSC2 under the control of a Flp-out *Act5C>CD2>Gal4* driver) and immune-precipitated using a Yki antibody [15]. To produce enough tissue despite TOR inhibition, discs were allowed to grow to a large size before a strong heat shock was used to excise the stop cassette within the *Act5C>CD2>Gal4* driver and initiate Gal4 expression in nearly all cells (Fig 5A and 5B; loss of magenta CD2 marks the *Act5C>Gal4* expressing tissue), with dissection of 200 discs per genotype 8–10 hr later (see Methods).

In control discs, as expected, Yki was enriched ~2–3-fold within the *2B2C diap1* element, using primers centered around the CATTCA motif to which Yki-Sd binds (relative to primers defining unrelated DNA sequences, as well as an IgG control treatment; Fig 5C). Knockdown of Yki (using the same *Act5C>CD2>Gal4* protocol to drive transient expression of a *UAS*.*yki* ^*RNAi*^ transgene) abolished this peak of enrichment, confirming its dependence on Yki levels and also validating the specificity of the antibody (S8A and S8B Fig). Strikingly, in experimental, TOR-inhibited discs, Yki enrichment at 2B2C was also diminished, by almost half (*p* < 0.05, *n =* 4 fully independent experimental replicates; Fig 5C; S1 Data). This reduction occurred despite the clear increase in nuclear Yki resulting from TOR inhibition (Fig 5A and 5B).

These data provide evidence that inhibiting endogenous TOR activity reduces the amount of Yki bound to a canonical Yki-Sd target gene enhancer, even as it causes an increase in Yki nuclear accumulation. Hence, we propose that TOR inhibition renders Yki subject to a phenomenon that we term “nuclear seclusion”, whereby Yki is mobilized and/or sequestered in the nucleus but hindered from accessing its target enhancer sequences. We posit that this mechanism accounts at least in part for the reduction in DIAP1 expression observed when TOR is inhibited (Fig 4B) and suggest that it likewise explains the loss of expression of other TOR-dependent Yki-Sd target loci for which defined enhancers have not yet been characterized (e.g., *fj*, *ex*; Fig 4F and 4H).

It is important to note that in most of our experiments, we have strongly reduced TOR signaling by coexpressing TOR^TED^+TSC1&2, to approximate, or at least approach, TOR loss-of function. These experiments reveal a basic requirement in wing cells for at least some level of TOR signaling in order to override nuclear seclusion of Yki and permit it access to drive transcription of target genes. However, we additionally propose that nuclear seclusion also operates within the normal physiological range of TOR activity as set by nutrient intake. First, weaker impairment of TOR signaling, by expression of TOR^TED^ alone, produces a wing size comparable to a moderately staved animal (Fig 1C). And yet it also leads to the two hallmarks of nuclear seclusion: increased Yki accumulation (S5D Fig), with concomitant reduction in expression of target genes (S5E Fig). Second, moderate reductions in TOR activity with RNAi transgenes directed against TOR and Rheb also reduce wing blade size within the range of normal trait scaling [5] (S5A–S5C Fig). Again, these manipulations cause detectable—albeit correspondingly much less pronounced—nuclear accumulation of Yki (S5F and S5H Fig) and reduced target gene expression (S5G and S5I Fig). Hence, while our assay of strong TOR inactivation (TOR^TED^+TSC1&2) gives the clearest observable nuclear accumulation, and is the basis for most of our mechanistic insights regarding Yki nuclear seclusion, we posit that this phenomenon operates beyond a simple baseline requirement for TOR signaling and is modulated by fluctuations of TOR signaling within the normal range. Furthermore, we note that because Yki is predominantly cytoplasmic in wing cells (Fig 3A), the small fraction of active, nuclear, Yki might be effectively secluded by quite modest reductions in TOR signaling (without leading to the profound nuclear accumulation witnessed when TOR is strongly impeded).

### TOR Inhibition Impedes Target Gene Access by Sd, in Addition to Yki

What mechanism mediates Yki nuclear seclusion in response to TOR inhibition? Given the similar effect of Sd overexpression on Yki localization and target gene transcription, we considered the possibility that the mechanism mediating Yki nuclear seclusion could be a pronounced increase in Sd protein level. However, Sd is already at high level throughout the developing wing, and TOR inhibition does not cause a further increase, as monitored by the expression of a fully functional GFP protein trap allele of the endogenous *sd* gene (*sd* ^*GFP*^; [49]) (S4J” Fig J; S8G and S8H Fig). Hence, we sought to assess two other possibilities: (i) that Yki, alone, is subject to nuclear seclusion, e.g., by being titrated away from enhancer-bound Sd, or (ii) that Sd, in addition to Yki, and perhaps in complex with it, is targeted for seclusion.

Using *sd* ^*GFP*^ wing discs and a GFP antibody, we assessed Sd-GFP enrichment at the *2B2C diap1* enhancer by ChIP. Like Yki, Sd-GFP is strongly enriched at *2B2C* in wild-type discs (Fig 5D). However, inhibiting TOR activity caused a reduction in Sd-GFP enrichment at *2B2C* to a similar degree as it does Yki enrichment (Fig 5C and 5D). Hence, it appears that TOR inhibition results in nuclear seclusion of Sd as well as Yki, rather than just Yki. One explanation for this observation is that Sd might need to be bound by Yki to bind *2B2C* —indeed, such a cooperative interaction appears to be required for enhancer binding by Sd in combination with Vg, another Sd transcriptional coactivator [50]. However, we observed that RNAi knockdown of Yki, which as expected results in reduced Yki binding at *2B2C*, has no effect on Sd enrichment (S8B Fig). That Sd binding to *2B2C* apparently does not depend on Yki argues against cooperative target site binding as a possibility. We therefore posit two possibilities: that Sd in complex with Yki is subject to nuclear seclusion, or, alternatively, that TOR inhibition secludes Sd in addition to, but independently of, Yki. If this second scenario were the case however, we may expect Sd in complex with transcriptional activators other than Yki to be influenced by seclusion. Conversely, expression of *vg*, an autoregulated target of Vg-Sd but not Yki-Sd complexes in wing cells, is not affected when TOR activity is inhibited (S4H and S4I Fig). Hence, we favor the view that nuclear seclusion acts on Sd in complex with Yki, although this additional facet of the mechanism remains to be confirmed.

### Nuclear Seclusion Is Mediated by Yki’s N-Terminus and WW Domains, But Not by Its Wts Phosphorylation Site

Yki is a modular protein with three regions that are conserved with vertebrate YAP and essential for normal function (Fig 5E): (i) a Wts phosphorylation site which, when phosphorylated, mediates cytoplasmic retention of Yki by 14-3-3 anchor proteins (ii) two WW domains that bind both Wts and Ex; and (iii) an N-terminal region, the Sd/TEAD binding domain (TBD), that mediates binding to Sd. Yki normally shuttles between the cytosol and nucleus in response to the state of phosphorylation at the Wts site [15–17], with phosphorylated and unphosphorylated forms accumulating, respectively, in the cytosol and nucleus. This shuttling is revealed by the build-up of nuclear Yki in wing cells following treatment with the nuclear export inhibitor Leptomycin B (S6D and S6E Fig; [51]). However, TOR inhibition is unlikely to cause abnormal Yki nuclear accumulation by reducing Wts-dependent phosphorylation of Yki, as this would mimic the consequence of Wts/Hpo inactivation [15–17], namely target gene induction rather than the observed repression. We therefore considered an alternative possibility that a different mechanism of Yki regulation, perhaps mediated by one or both of the other conserved Yki motifs, might be at play.

To shed further light on the mechanism of nuclear seclusion, we generated *Tubα1*. *yki* ^*GFP*^ transgenes that express GFP-tagged forms of Yki at near-physiological levels (S8C Fig; S1 Data) and analyzed the consequences of mutating each of the three conserved functional domains on the subcellular accumulation of Yki. TOR inhibition caused nuclear accumulation of wild-type *Yki* ^*GFP*^ as expected (Fig 5F). However, introducing a mutation, P88L, in the N-terminal TBD that diminishes binding to Sd [19] prevented nuclear accumulation in TOR-inhibited cells (Fig 5G). This block to nuclear accumulation appears to be specific to the loss of TOR pathway activity, as Yki^GFP-P88L^ protein accumulates in the nuclei of *wts* ^*—*^wing cells that have normal TOR pathway activity (Fig 5K), and the same is true for Yki^GFP-P88L^ protein that is additionally mutated for the Wts phosphorylation sites in otherwise *wild type* wing cells (Fig 5 and 5J). Likewise, mutating Yki’s two WW domains prevents nuclear accumulation following TOR inhibition (Fig 5H), even though the WW domains are known not to interfere with nuclear accumulation following Wts/Hpo pathway inactivation [52]. Thus, it appears that the Wts-phosphorylation site governs whether Yki has access to the nucleus: only Yki in which this domain is not phosphorylated by Wts can enter. In contrast, interactions mediated by the TBD and WW domains appear to determine whether any unphosphorylated Yki that gains access to the nucleus will be secluded there when TOR activity is inhibited. Accordingly, the abnormal nuclear accumulation of Yki resulting from TOR inhibition should represent a build-up of unphosphorylated Yki, even when Wts is present, as any unphosphorylated Yki that enters the nucleus will get trapped there. Consistent with this prediction, we observed a general reduction in phosphorylated Yki-S168 levels following TOR inhibition (S8D Fig). (Note that although we interpret this as a consequence of nuclear seclusion, we cannot rule out an additional, independent effect of TOR inhibition in dephosphorylating Yki. However, we note that if such an activity occurs, it has seemingly negligible function consequences for Yki activity; dephosphorylated Yki should increase target gene expression, but our data show that blocking TOR strongly reduces Yki target gene expression).

Based on these data, we infer that low levels of InR/TOR signaling activity result in the induction or function of a presently unidentified Nuclear Secluding Factor (NSF) that acts in wing cells to sequester Yki in the nucleus and to divert Yki-Sd from its target genes. The putative NSF might interact with Yki directly, via Yki’s N-terminus and/or WW domains and cause Yki nuclear seclusion by both increasing the binding of Yki to Sd and preventing the resulting NSF-Yki-Sd complex from accessing Yki-Sd target genes. Accordingly, NSF action would result in enhanced Yki nuclear accumulation and reduced Yki-Sd target gene expression. This mode of Yki regulation acts independently of the Hpo-Wts mediated cytosolic tethering of Yki to limit target gene expression. Indeed, even in Hpo pathway mutant tissue (e.g., loss of Ex, or Wts), where Yki is constitutively dephosphorylated, TOR inhibition still leads to increased Yki nuclear accumulation (S4E–S4G Fig) and decreased Yki target gene transcription (S4C–S4D Fig; S8E and S8F Fig).

### TOR Regulates Yki Nuclear Seclusion and Cellular Metabolism in Parallel to Control Wing Growth

The requirement for InR/TOR signaling activity to allow Yki to escape nuclear seclusion is revealed by genetic manipulations that compromise InR/TOR pathway activity and cause Yki to accumulate nonproductively in the nucleus. Importantly however, we do not think that TOR signaling promotes Yki-driven wing growth solely by this mechanism. Instead, we posit that this mechanism operates in parallel with other outputs of InR/TOR signaling that regulate a range of basic metabolic processes that are necessary, independently, for cell growth as well as rate-limiting for cell proliferation [5,10,53]. A clear demonstration of this parallel requirement for InR/TOR signaling is the observation that Yki-driven overgrowth of the wing (caused by removal of *wts*) could be suppressed by removal of S6-Kinase (dS6K), a TOR target that catalyses cap-dependent mRNA translation (S7A–S7D Fig; S1 Data), but which is not itself involved in nuclear seclusion (S3G, S3H and S3J Fig).

Hence, while TOR/InR activity is needed to override Yki nuclear seclusion, it is not sufficient to promote proliferative growth in the absence of concomitant activation of canonical metabolic targets like dS6K. Coupled to our reciprocal finding above, that TOR activity is unable to promote cell proliferation without Yki (Fig 2F–2L), we conclude that neither output of TOR is sufficient. Rather, both outputs are required, first to unleash Yki from nuclear seclusion, and second to provide the metabolic wherewithal for Yki target genes to promote growth. By concomitantly compromising both outputs, nutritional deprivation coordinates Yki target gene expression with decreased metabolic capacity to reduce the growth rate of the wing.

### Superphysiological TOR Activity Triggers a Regulatory Feedback That Circumvents Wing Overgrowth

**Fig 6:**
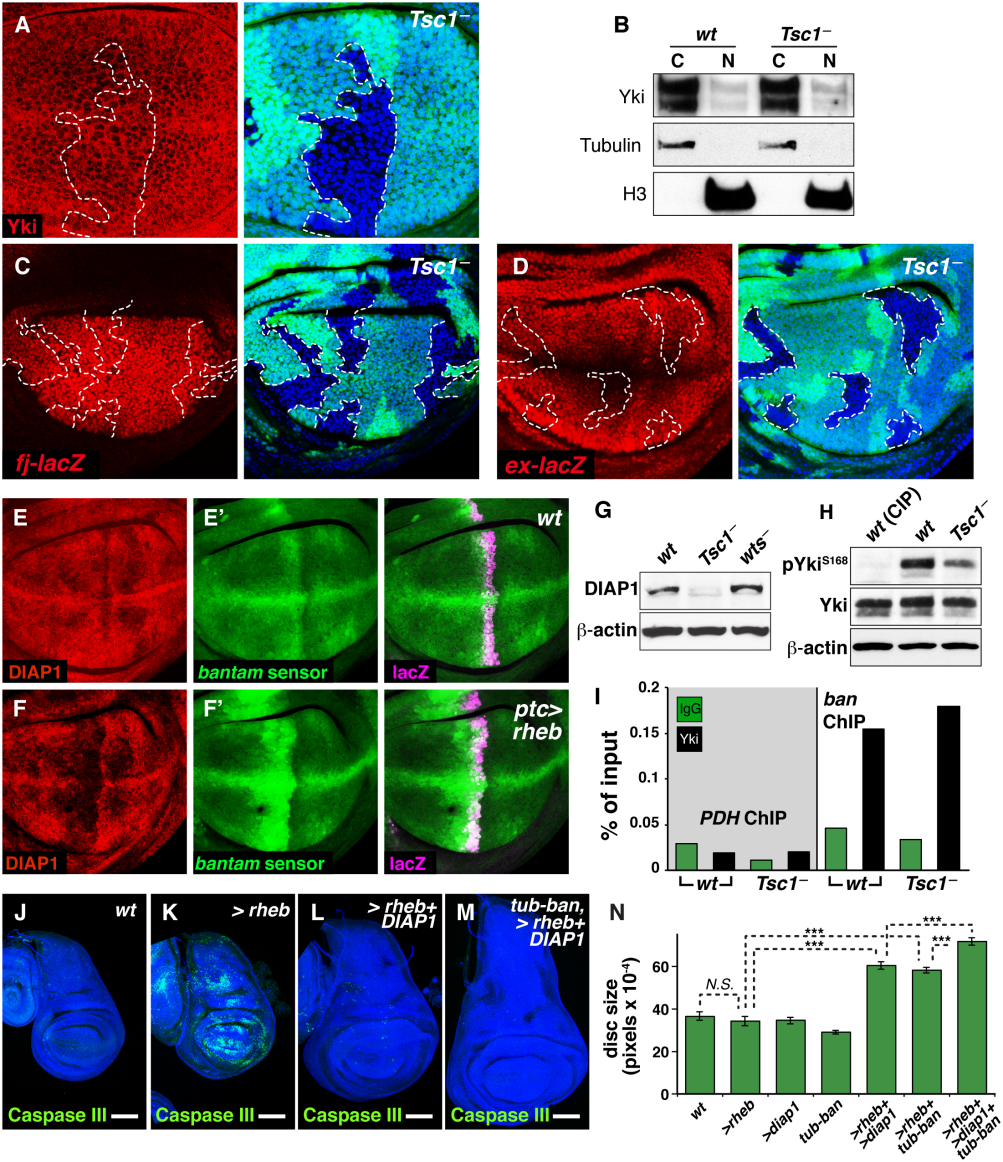
Superphysiological TOR activity causes excess, Yki-independent growth that is offset by a negative feedback that down-regulates the anti apoptic factors DIAP and *bantam*. (**A**) Wing disc carrying a clone of *Tsc1* ^*Q87X*^ mutant cells (marked black by absence of GFP, green, and outlined with a dashed white line; counterstained with Hoechst, blue). Yki accumulation (red), imaged at the nuclear plane, is unchanged in the clone. **(B)** Yki nucleocytoplasmic distribution is similar in wild-type and *Tsc1* ^—^discs. Effective separation of cell fractions was confirmed by Tubulin (cytoplasm) and Histone 3 (nucleus). (**C, D**) Wing discs carrying *Tsc1* ^*Q87X*^ mutant clones (marked and imaged as in **A**): expression of the Yki target genes *fj-lacZ* (**C**) and *ex-lacZ* (**D**) (red) is not affected. (**E, F**) Compared to wild type discs (**E**), expression of Rheb in a stripe under *ptc*.*GAL4* control (**F**) causes a reduction in DIAP accumulation (red), as well as *bantam* micro-RNA activity, the latter indicated by relief of repression of a *bantam-GFP* sensor (green) [54]; peak activity of the *ptc*.*Gal4* driver is indicated by expression of β-galactosidase from a *UAS*.*lacZ* transgene, magenta). (**G**) Protein extracts of wild type, *Tsc1* ^*Q87X*^/*Tsc1* ^*PA23*^, and homozygous *wts* ^*P2*^ (positive control) discs probed for DIAP1 protein reveal that DIAP1 is strongly reduced in *Tsc1* ^—^discs. β-actin was used as a loading control. (**H**) Phospho-Yki S168 levels *Tsc1* ^—^homozygous mutants discs are not elevated (and are in fact mildly reduced) compared to *wild type* control discs (total Yki and β-actin were used as loading controls; CIP treatment was used to ensure the correct product was being observed). **(I)** *Tsc1* ^*—*^discs do not show a reduction in Yki enrichment at a Yki responsive enhancer in the *bantam* locus compared to wild-type discs. IgG mock IP and enrichment at the PDH (*pyruvate dehydrogenase*) locus were included as controls. (**J, K)** *hdc-GAL4* wing discs that either do (**K**, experimental) or do not (**J**, control) express a *UAS*.*rheb* transgene, labelled for active caspase III (green): both discs are approximately the same size (counterstained with Hoechst, blue), but the experimental disc shows pronounced Caspase activity in contrast to the control. (**L–N**) Coexpressing a *UAS*.*diap* transgene together with *UAS*.*rheb* (**L**) prevents cell death caused by expression of *UAS*.*rheb* (**K**) and results in tissue hyperplasia, as indicated by the increase in disc size. Hyperplasia is further increased by the addition of *bantam* expression under the direct control of the *Tubα1* promoter (**M**). (**N**) Quantification of disc sizes. Error bars are Standard Error of the Mean and asterisks denote significances from *t* tests (* = *p* < 0.05, ** = *p* < 0.01, *** = *p* <0.001, *n*. *s*. = not significant). *n* = 20 (*wt*), 24 (*Rheb*), 18 (*diap1*), 10 (*tub-ban*), 20 (*rheb+diap1*), 15 (*rheb+tub-ban*), 22 (*rheb+diap1+tub-ban*).

Our results describe how inhibiting TOR signaling reduces Yki function as part of the mechanism that might scale wing growth downwards in response to nutritional deprivation. To further elucidate how TOR and Yki might function together to control wing size, we asked whether superphysiological TOR activity might alter Yki localization and/or activity to scale wing size upwards. However, we were unable to detect any such changes in Yki using either of two well-established approaches to overactivate the InR/TOR signaling in vivo. Specifically, overactivating TOR by removing the negative regulator TSC1 in wing cells did not appear to alter the nuclear-cytoplasmic distribution of Yki as assayed either by immunofluorescence in clones of *Tsc1* ^—^mutant cells (Fig 6A) or by quantitating the nuclear–cytoplasmic ratio of Yki in fractionated whole wing discs from entirely *Tsc1* ^—^mutant larvae (Fig 6B). Similarly, we tested whether clonal removal of TSC1, or alternatively, clonal overexpression of the positive regulator Rheb, affects the expression of any of several Yki target genes, and again, observed no differences (Fig 6C and 6D; S9A and S9B Fig). Hence, superphysiological gains in TOR activity do not appear to influence either Yki localization or activity, suggesting that peak endogenous TOR signaling is sufficient to fully override Yki nuclear seclusion as well as any other InR/TOR-dependent constraints on Yki coactivator function.

Nevertheless, manipulations that generate superphysiological levels of TOR activity do reveal a further facet of the TOR-Yki relationship in the wing. We noticed that despite strongly elevating TOR signaling in wing discs (Fig 2E), loss of *Tsc1* activity failed to alter overall wing disc size (S9C and S9D Fig). Instead, it appears to cause excess growth that is counterbalanced by apoptosis (S9C and S9D Fig). The same was true of discs overexpressing *rheb* in all cells under the control of the imaginal disc-specific *headcase*.*GAL4* driver (*hdc*.*G4*; Fig 6J, 6K and 6N; S1 Data), which produces an equivalent level of TOR activation to loss of *Tsc1* (compare phospho-S6K levels in S7I Fig to those of *Tsc1* ^*—*^discs in Fig 2E). This increase in apoptosis can be attributed to a repressive effect of superactivation of TOR on the expression of DIAP1 protein and the *bantam* microRNA (Fig 6E–6G; S9F and S9F’ Fig), both targets of Yki regulation that have antiapoptotic activity [55]. However, the repression of DIAP1 and *bantam* does not appear to be due to enhanced Wts kinase activity, as *Tsc1* ^—^discs showed a mild reduction rather than an increase in phospho-Yki S168 levels (Fig 6H). Moreover, the effects on DIAP1 and *bantam* are seemingly Yki-independent, since the repression of DIAP1 appears to be posttranscriptional (S9A and S9B Fig), and ChIP experiments indicate that Yki binding to its target enhancer in the *bantam* gene is not reduced in *Tsc1* ^*—*^wing discs (Fig 6I) [56]. In principle, DIAP repression could be a secondary consequence of *bantam* repression, since DIAP1 protein is negatively regulated by the Head involution defective protein (Hid) [57], a *bantam* target [58]. However, we failed to observe any effect of overexpressing or removing *bantam* on DIAP1 protein levels (S9G and S9H Fig), from which it appears that TOR superactivation does not act via *bantam* to repress DIAP. Thus, TOR overactivation appears to cause cell death independently of the Wts/Hpo/Yki pathway, possibly mediated by reduced expression of both DIAP1 and *bantam*.

To gauge if abnormally high levels of TOR activity indeed repress DIAP and *bantam* to offset tissue overgrowth, these gene products were resupplied to discs overexpressing *rheb* under *hdc*.*GAL4* control. Overexpression of *diap1*, alone, under *hdc*.*GAL4* control produced no significant effect on wing disc size (Fig 6N; S1 Data). However, co-overexpression of *diap1* together with *rheb* fully suppressed cell death that would otherwise result from the overexpression of *rheb* alone (Fig 6L), and disc size increased by 77% (Fig 6N; S1 Data). To resupply *bantam*, GAL4-dependent transgene expression was not used, since *bantam* overexpression itself induces growth [58,59], rendering synergistic outcomes difficult to interpret. Instead, a transgene was constructed that expresses GFP at low levels under the direct control of the *Tubα1* promoter, with the 3’UTR containing a minimal 100 bp genomic *bantam* fragment that includes the 21 bp *bantam* microRNA [58]. The resulting *Tubα1*.GFP-*bantam* transgene does not itself stimulate overgrowth (Fig 6N; S1 Data) but produces enough *bantam* to rescue a homozygous null *bantam* animal to adulthood, producing a normally sized and patterned animal (S9O and S9P Fig). This suggests that a single copy of *Tubα1*.GFP-*bantam* produces a level of *bantam* activity comparable to homozygosity for the endogenous *bantam* gene. Strikingly, when introduced as a single copy into the *hdc*.*G4>UAS*.*rheb* background, disc size increased 70% (Fig 6N; S9N Fig; S1 Data), similar to the effect of co-overexpressing DIAP with *rheb*, although the addition of the *Tubα1*.GFP-*bantam* did not fully suppress cell death (compare Caspase staining in S9L and S9M Fig). Finally, combining co-overexpression of *rheb* and *diap1* with *Tubα1*.GFP-*bantam* led to still more growth, with the wing disc more than doubling in size (110% larger; Fig 6H and 6N; S1 Data).

In sum, the effects of reintroducing DIAP1 and *bantam* reveal a novel effect of superphysiological TOR activity: the repression two key Yki target genes, albeit by Yki-independent mechanisms, to induce cell death and counterbalance the excessive tissue growth that would otherwise result from TOR hyperactivity. We suggest that this regulatory circuit normally safeguards against organ overgrowth and overriding it exposes a hidden capacity of superphysiological TOR signaling to stimulate tissue hyperplasia.

## Discussion

Organ size depends on organ-intrinsic mechanisms that couple cell proliferation to the specification of pattern, as well as on external signals such as nutrients that scale overall body size [6–9]. Our study reveals one way in which these two distinct developmental phenomena can be integrated in the developing *Drosophila* wing, via a circuit linking nutrient-controlled TOR (and upstream InR) activity to Yki target gene access. A key finding is our discovery that, in addition to the well-known cytoplasmic anchoring mechanism employed by the Wts/Hpo tumour suppressor pathway to constrain Yki nuclear access, the InR/TOR pathway operates by a wholly different mechanism to allow nuclear Yki to activate its target genes. Inhibiting each pathway results in the accumulation of Yki in the nucleus, but with opposite effect. Nuclear Yki resulting from reduced Wts/Hpo activity drives Yki target gene expression and promotes growth. But, strikingly and counterintuitively, nuclear Yki resulting from reduced InR/TOR activity causes a loss of target gene access and impedes growth.

**Fig 7:**
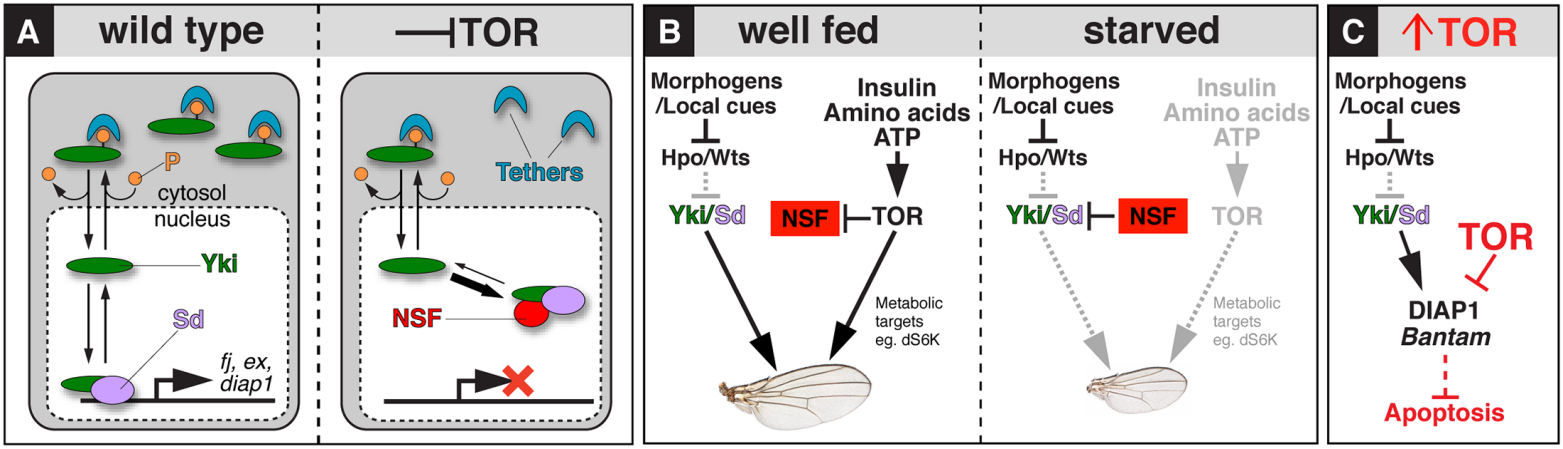
Integrating Yki-dependent wing growth with InR/TOR pathway activity. (**A**) Under normal physiological conditions (left panel), Yki shuttles between the nucleus and cytoplasm in response to phosphorylation (P) by Wts, which targets Yki to cytosolic tethers. Wing disc intrinsic signals (e.g., the morphogens Dpp and Wg) drive wing growth by down-regulating Wts activity, allowing a small proportion of Yki to escape phosphorylation-dependent tethering, enter the nucleus, and bind to its growth-promoting target genes in complex with Sd. Inhibiting the InR/TOR pathway results in up-regulation or activation of a putative NSF that sequesters unphosphorylated Yki in the nucleus and impedes binding of the Yki-Sd complex to its target genes, reducing Yki-Sd dependent growth. (**B**) Proposed integration of InR/TOR and Wts/Yki signaling to scale wing growth. In well-fed animals (left panel), TOR activation by wing disc extrinsic, nutrient-dependent signals facilitates Yki-dependent tissue growth by two parallel means: (i) by inhibiting NSF and thereby potentiating Yki nuclear access and target gene expression, and (ii), by up-regulating cell physiological functions such as dS6-Kinase and possibly many others, to match growth potential to the level of Yki target gene activity. Reductions in InR/TOR activity diminish both outputs, leading to reduced cell proliferation and tissue growth. (**C**) Excess growth caused by superphysiological activation of TOR is offset by Yki-independent down-regulation of the antiapoptotic factors DIAP1 and *bantam*, promoting cell death and safeguarding the developing wing against hyperplasia.

Based on our results, and as summarised in Fig 7A, we propose that reduced TOR signaling causes Yki to bind a secluding factor that sequesters Yki in the nucleus but diverts it from acting in complex with Sd to bind target loci, thereby reducing gene transcription. We posit that, by combining TOR-dependent relief from Yki nuclear seclusion with canonical Wts/Hpo phosphoregulation of Yki nuclear access, wing cells are able to integrate nutrient levels (via TOR) with patterning/morphogenetic inputs (via Wts/Hpo) to achieve a level of Yki-Sd activity that matches metabolic capacity and scales wing size appropriately. Elucidating this mechanism further will depend on identifying the proposed NSF and determining how its action is mediated by Yki’s N-terminus and WW domains. NSF may bind Yki that shuttles into the nucleus, titrating it away from target genes; one possibility, raised by a reviewer, is that NSF acts by modifying Yki in some way, stabilizing the protein and preventing its degradation (thereby contributing to its observed accumulation in the nucleus) with a modification that blocks it from accessing target loci.

A model for how TOR and Wts/Hpo inputs are integrated during normal *Drosophila* wing growth is depicted in Fig 7B. When the native TOR pathway is signaling at its peak, the level of Wts/Hpo activity dictates the amount of unphosphorylated Yki that is free to enter the nucleus. TOR activity liberates the Yki-Sd complex from a default state of nuclear seclusion, permitting nuclear Yki-Sd to access and transcriptionally activate loci that promote proliferative growth. In parallel, TOR also stimulates cellular metabolic processes such as protein translation (mediated by targets such as dS6K), which are preconditions for tissue growth. Both outputs of TOR are required for wing cells to divide and gain mass under Yki-Sd control. Given the central role of TOR signaling in nutrient sensing, [5,10], we further propose that this circuit may contribute to the capacity of the developing wing to scale downwards under conditions of nutrient deprivation. Specifically, under nutrient-limiting conditions, we suggest that the consequent reduction in TOR activity renders the Yki-Sd complex subject to nuclear seclusion, thereby decreasing transcription of Yki-Sd target genes that promote growth. Reduced TOR activity would also diminish metabolism, matching metabolic capacity with the reduced activity of Yki-Sd target genes. Such a circuit would provide a sensitive means for developing tissues to integrate systemic and local inputs into growth. Our finding that excess activity of TOR appears to cause excess growth that is compensated by cell death via reduced DIAP1/*bantam* expression (Fig 7C) reveals that the circuit linking TOR and Yki is additionally stabilized by negative feedback, constraining organ growth in the event of erroneous, superphysiological TOR activity. Hence, a previously unknown facet of nutrient sensing by TOR is the incorporation of a self-limiting tumor suppression mechanism.

While our study delineates a circuit that integrates TOR and Yki activity during *Drosophila* wing growth, we note that it is only in this tissue that we find unequivocal evidence for nuclear seclusion of Yki, the means by which we posit this integration is achieved. Blocking TOR signaling in two other imaginal disc types, the eye and leg, does not appear to affect either Yki localization or target gene expression (S8I–S8K Fig). The same is also true even for other cell populations within the wing imaginal disc, notably those giving rise to the wing hinge and body wall. Hence, nuclear seclusion of Yki does not appear to be a general consequence of inhibiting TOR activity, and may occur primarily (or possibly only) in presumptive wing cells.

Intriguingly, the wing primordium is unique amongst imaginal disc tissues in expressing high levels of Sd under the control of Vg, a transcriptional coactivator that forms a complex with Sd to “select” the wing state. Although Yki-Sd complexes regulate growth control downstream of the Wts/Hpo pathway in the other *Drosophila* imaginal discs, Sd is expressed in these other tissues at tonic, low levels. Further, in the wing, Vg-Sd and Yki-Sd coexist, regulating distinct cohorts of target genes, and our present evidence suggests that inhibiting TOR does not alter the capacity of the Vg-Sd complex to gain access to, or activate, its distinct set of target genes (e.g., *vg* and *sd* itself). Hence, nuclear seclusion of Yki may represent an organ-specific mechanism to integrate nutrient-regulated InR/TOR and morphogen-dependent Wts/Hpo activities under conditions in which Sd plays a major, independent role in specifying cell state. It remains possible that TOR activity regulates nuclear seclusion of Yki to control growth in other tissues, and possibly in other animals, albeit in complex with other transcriptional effectors and primarily through the control of target gene access rather than nuclear access. Alternatively, TOR may limit growth in these other systems exclusively through its regulation of cellular metabolism, without additionally modulating the level of Yki activity. Studying how global chromatin occupancy by Yki changes in response to TOR pathway activity, and varies by tissue type, will be needed to evaluate these scenarios.

To our knowledge, nuclear seclusion represents a novel paradigm of transcription factor regulation. Although experimental overexpression of nuclear Yki cofactors (e.g., Sd [18,52]) can exert similar effects on Yki localization and target gene expression, thus far it has not been demonstrated that such a mechanism functions as a biologically relevant mode of transcription factor regulation operating in vivo. Presently, the identity of the factor mediating Yki nuclear seclusion is unknown. However, in both yeast [60] and mammals [61,62], mTOR functions as a classical kinase that phosphorylates diverse substrates, leading to their retention in the cytoplasm. We hypothesize that our proposed NSF is one such substrate. A potential avenue of future exploration will be systematically testing how TOR inhibition influences the increasing number of identified nuclear modulators of Yki—some of which, like Sd, affect Yki localization and function when overexpressed (e.g., Hipk [63,64], MASK [65,66] and Tgi [48,67]). As we have demonstrated for the regulation of Yki by TOR, nuclear seclusion offers a complementary mechanism for controlling transcription factors that are already subject to phosphorylation-dependent partitioning between the cytosol and nucleus. Nuclear seclusion may therefore be used more widely to achieve signal integration, both during development as well as in mature tissues.

## Methods

### *Drosophila* Genetics

The following mutant alleles, Flp Recombination Targets (FRTs), *UAS* transgenes, *Gal4* drivers and reporter transgenes were used (source indicated in parentheses; see Flybase http://flybase.org/ for details).

#### Mutations and FRTS

*Tor* ^*ΔP*^ *FRT40A* (T. Neufeld), *ex* ^*e1*^ *FRT40A* (G. Halder), *FRT82B rheb* ^*AV4*^ (Bloomington), *bantam* ^*Δ1*^ *FRT2A*, *FRT82B wts* ^*X1*^, *FRT82B akt* ^*q*^ (S. Cohen), *FRT82B foxo* ^*25*^ (E. Hafen), *FRT42D yki* ^*B5*^ (D. Pan), *pten* ^*1*^ *FRT40A* (C. Wilson), *pten* ^*dj189*^ *FRT40A* (D. Pan), *FRT82B Tsc1* ^*Q87X*^ (I. Hariharan), *FRT82B Tsc1* ^*PA23*^ (G. Halder), *wts* ^*p2*^ (G. Halder), *ds6k* ^*I–1*^ (E. Hafen), *Thor* ^*2*^ and *sd* ^*GFP*^ (Bloomington).

#### UAS transgenes

*UAS-p35* (B. Hay), *UAS- ΔP60* (S. Leevers), *UAS-Yki* (D. Pan), *UAS-Dp110* (S. Leevers), *UAS-Rheb* (B. Edgar), *UAS-TSC1+2* (I. Hariharan), *UAS-Tor* ^*TED*^ (T. Neufeld), *UAS-TorRNAi* (P{TRiP.HMS01114}attP2; Bloomington), *UAS-RhebRNAi* (P{TRiP.HMS00923}attP2; Bloomington), *UAS*-*Yki* ^*RNAi*^ (VDRC), *UAS-HA-Sd* (J. Jiang), *UAS*.*GFP*, and *UAS*.*GFP-NLS*.

#### Gal4 drivers

*nubbin*.*GAL4*, *tubulin*.*GAL4*, *headcase*.*GAL4* (C. Lee) *act>CD2>GAL4*, *dpp*.*GAL4*, and *ptc*.*GAL4*.

#### Reporter transgenes

*dll-lacZ*, *ex* ^*e1*^ *-lacZ* (*ex* ^*e1*^, G. Halder), *fj-lacZ*, *diap* ^*2B2C*^ *-lacZ* (D. Pan), *diap* ^*j5c8*^-*lacZ*, and the *bantam* sensor [58] were used.

For generation of mutant heads, the *EGUF/Hid* technique was used [68]. For generation of negatively marked mutant clones, *hsp70*.*flp; mut FRT/ubi*.*GFP FRT* larvae (“*mut*” standing for the mutation(s) of interest) were heat shocked to induce by Flp-mediated mitotic recombination. Similarly, positively marked MARCM clones were generated by heat shocking *hsp70*.*flp UAS*.*GFP-NLS Tuba1*.*Gal4 / Y* or *hsp70*.*flp; mut FRT* / *Tuba1*.*GAL80 FRT* larvae. For *Minute* ^*+*^ clones on 3R, an *FRT ubi*.*GFP M(3)95A* chromosome was used.

### Quantification of Wing and Clone Sizes and Cell Numbers

All measurements were made blind, without knowledge of the genotype. When performing multiple *t* tests involving the same treatments, Bonferroni-corrected *p*-values were used to assess the level of significance. Clone and wing sizes were measured in Adobe Photoshop using the “lasso” tool, and cell numbers counted using the “count” tool. For whole wing cell number estimates, setae were counted within a 170 x 170 pixel box within the posterior intervein region. Total wing cell number = (wing size in pixels / (170 x 170)) x cell number in 170 x 170 pixel box.

### Immunohistochemistry and Scanning Electron Microscopy

Discs were dissected, fixed in 4% PFA and stained with the following antibodies: rabbit anti-Phosho-AKT (S505) (1:200, Cell Signalling Technology), rabbit anti-Distal-Less (1:3,000), rabbit anti-Yki (1:500; D. Pan), mouse anti-DIAP1 (1:500), rabbit anti-ß-galactosidase (1:6,000; Cappel), mouse anti-Ptc (1:150; DHSB), mouse anti-Armadillo (1:10; DSHB), guinea pig anti-Distal-less (1:3,000; R. Mann), mouse anti-CD2 (1:500; BD Biosciences). Alexa Fluor secondaries (Invitrogen) were used, and Hoechst was used to label nuclei. A Leica SP5 confocal microscope was used to capture all immunofluorescence images. For SEM, adult heads created by the *EGUF/Hid* technique were dissected into 70% ethanol, dried with an AUTOSAMDRI critical point drier, gold coated and imaged using the Hitachi S-3400N Variable Pressure SEM installed at the Microscopy Imaging Lab, University of Toronto.

### Protein Blotting

For standard blotting of total wing protein, ~80 wing discs/lane were dissected from wandering third instar larvae into laemmli sample buffer supplemented with DTT, Complete Mini EDTA-Free protease inhibitor (“CPI” Roche) and Phosphatase Inhibitor Cocktail III (Calbiochem). Samples were boiled for 10 min and protein was quantified using the Pierce 660nm assay. Equal amounts of protein were loaded per lane. For fractionation of wing disc cells, ~80 wing discs were dissected per genotype, and washed twice with ice cold PBS+CPI. Discs were then washed briefly in 10μl hypotonic buffer (10 mM HEPES + 1.5 mM MgCl_2_ + 10 mM KCL + CPI), centrifuged (1,500 rpm, 5 min, 4°C), and incubated in 30 μl hypotonic buffer on ice for 10 min. Discs in buffer were then dounced ~30 times in a glass homogeniser. Nuclei were separated by centrifuging (15 min, 3,750 rpm, 4°C) and removing the cytoplasmic fraction. Nuclei were washed three times in hypotonic buffer (spinning down and resuspending each time), and finally resuspended in 30 μl hypotonic buffer. 40μl laemmli sample buffer + DTT + CPI was added to both nuclear and cytoplasmic fractions, which were then boiled for 10 minutes and quantified using the Pierce 660 nm assay. Equal amounts of nuclear and cytoplasmic protein were loaded per lane. Antibodies used were rabbit anti-Phospho-*Drosophila* Akt S505 (1:500; Cell Signalling), anti-Akt (1:1,000; Cell Signalling), anti-Phospho-*Drosophila* p70 S6 Kinase T398 (1:500; Cell Signalling), rabbit anti-Yki (1:1,000, K. Irvine), rabbit anti-phospho Yki S168 (1:1,000; D. Pan), mouse anti-DIAP1 (1:1,000; B. Hay), mouse anti-ß-actin (1:10,000; Abcam), rabbit anti-Histone 3 (1:10,000) and mouse anti-ß-tubulin (1:500; DSHB). HRP-conjugated secondaries (Invitrogen) were used, and blots were developed with an ECL Plus detection kit (Amersham).

### Chromatin IP

For ChIP of Yki and Sd-GFP at the 2B2C locus, the following procedure was undertaken four times to generate four fully independent experimental replicates. Female larvae of the experimental genotype (*sd-GFP/y w hs-flp*; *UAS-Tor* ^*TED*^ */+; act*>*CD2*>*GAL4/Tsc1&2*), negative control female larvae (*sd-GFP/y w hs-flp; act*>*CD2*>*GAL4/+*) or *Yki* ^*RNAi*^ control (*sd-GFP/y w hs-flp; UAS-Yki* ^*RNAi*^ */+; act*>*CD2*>*GAL4/+*) were allowed to reach the late larval stage before a 1 hr/37°C heatshock. This caused widespread >*CD2*> cassette excision leading to an extremely high frequency of clones expressing TOR^TED^ and TSC1 + 2, or Yki^RNAi^. Eight to ten hours later, 200 experimental and control larvae were into PBS + Complete Mini EDTA-Free protease inhibitor (denoted “CPI” Roche). Following the protocol of Estella et al. [69], batches of 25–30 larvae/tube were fixed (25 min; 1.8% formaldehyde in 50 mM HEPES + 1 mM EDTA + 0.5 mM EGTA + 100 mM NaCl + CPI), quenched (6 min; 0.125 M glycine in PBS + 0.01% Triton) and passed through a buffer series to prepare nuclei for lysis (2 washes 10 min Buffer A: 10 mM HEPES + 10 mM EDTA + 0.5 mM + EGTA + 0.25% Triton + CPI, 2 washes 10 min Buffer B: 10 mM HEPES + 200 mM NaCl + 1 mM EDTA + 0.5 mM EGTA + 0.01% Triton + CPI). Individual wing discs were then dissected from larvae and placed in sonication buffer (10 mM HEPES + 1 mM EDTA + 0.5 mM EGTA + CPI), and sonicated on ice. For each genotype, 10% of the fresh chromatin was removed for the input control, and the remainder was split into two samples, each diluted 1:1 with 2xRIPA buffer (280 nM NaCl + 20 mM HEPES + 2 mM EDTA + 2% glycerol + 2% Triton + 0.2% Sodium Deoxycholate). Samples were incubated overnight with either anti-Yki (1:315; D. Pan), anti-GFP (1:300; molecular probes) or anti-Rabbit IgG control (Cell Signalling Technology) antibodies. Antibody-bound chromatin was pulled down for 3–4 hr with Protein A Agarose (Roche), washed several times in 1xRIPA and once in TE. Chromatin was then eluted twice with elution buffer (50 ul 1% SDS, 0.1 M NaHCO_3_), once at room temperature and once at 55°C. Eluted chromatin was placed at 65°C to reverse crosslinks. Following a 3 hr treatment with Proteinase K, ChIP DNA was extracted with phenol/chloroform, ethanol precipitated and resuspended in TE. Quantitative real time PCR was performed using HotStart-IT SYBR Green qPCR Master Mix (2x) and an Applied Biosystems 7300 RT-PCR machine. For both genotypes from each of the three experimental replicates, DNA was amplified from total input control (serially diluted to avoid saturating the reaction), IgG-, GFP- and Yki-precipitated treatments, using primers for the 2B2C region of *diap1* (2B2CF3 5’-AGAAAACTCGAAAGGCAGCTC/2B2CR3 5’-CCAAAACCAAACCAACGAAC) and a control locus (*pyruvate dehydrogenase*; PDH_F 5’-CGGAAGTGAAGCTGACCAAG/PDH_R 5’-GTAGGTCCATCCGTGGA CAC). The Δ^ct^ method was used to calculate the degree of enrichment in each treatment relative to the input control DNA.

For ChIP at the *bantam* locus, chromatin was prepared from ~150 *wild type* and *Tsc1* ^*Q87X*^/*Tsc1* ^*PA23*^ mutant wing discs, and subjected to pulldown with ant-Yki or IgG control antibodies as explained above. An ~7 kb region around the microRNA was screened for Yki enrichment, and a strong peak was determined within a region 1,861–1,621 bp 5’ to the 21 bp hairpin, corresponding to positions 640419–640646 on chromosome 3L of the April 2006 assembly of the *Drosophila melanogaster* genome (BDGP R5/dm3). This region was targeted in qPCR using the primers: bantam3F 5’-CGGGAACAGTCATAAAAGTTG C/bantam3R 5’-CTTTGCCTGTTCTGCCAT CC. Note that this region lies inside the C12 enhancer region bound by Yki identified by Oh and Irvine [56].

### Transgene Construction

#### *tubulin-Yki-GFP* alleles

A full-length *yki* cDNA was amplified with KpnI and SpeI linkers, and C-terminally tagged with SpeI-EGFP-XbaI. Site directed mutagenesis was used to create Yki^P88L^, Yki^S111A S168A S250A^ (Yki^3S->A^), and Yki^W292A P295A W361A P364A^ (Yki ^ΔWW^), and combinations thereof. Yki-GFP alleles were inserted as KpnI-XbaI fragments into *p(Tuba1>DsRed*, *y2>)ATTB*, where > *DsRed*, *y* ^*2*^> is a flip-out cassette containing DsRed and *yellow* ^*2*^ markers. Transgenic animals were generated with phiC31 Integrase-mediated recombination, by transforming a *Drosophila* stock containing an ATTP landing site at 25C7 [70]. Excision of the intervening >*ds-Red*, *y* ^*2*^ *+*> cassette leads to ubiquitous, low level expression of Yki-GFP from the *alpha*-*tubulin* promoter.

#### tubulin-bantam

EGFP with a 3’UTR containing a 100 bp fragment encompassing the *bantam* hairpin [58] was inserted as a KpnI-XbaI fragment into p(*Tuba1>DsRed*, *y* ^*2*^ *>*)AttB, and the construct was inserted into the genome at 25C7. The *>DsRed*, *y* ^*2*^ *>* cassette was excised to generate *Tuba1>ban*. This flipped out form was introduced into the null *ban* ^Δ1^ homozygous background to assess rescue.

### Quantification of Transgenic Yki expression

To quantify Yki expression from the *tub-Yki-GFP* transgene, the cassette was removed in clones, and discs were stained for total Yki. The staining intensities of five *wild type* regions and five regions of clonal tissue were quantified in ImageJ and averaged. The ratio between clonal and *wild type* tissue was calculated as 1.69.

## Supporting Information

S1 Data Raw numerical values for quantification of wing, clone and cell sizes, chromatin immunoprecipitation, and fluorescence intensity measurements. (XLSX)

S1 Fig TOR inhibition blocks Yki-driven overgrowth of the wing and head. **(A–D)** Late third instar wing discs with mutant clones marked negatively by absence of GFP (green; *wild type* sibling clones appear as bright green). (**A**) *wild type* (control) (**B**) *rheb* ^*AV4*^, (**C**) *wts* ^*X1*^, (**D**) *rheb* ^*AV4*^ *wts* ^*X1*^. Note that in **b** and **d**, mutant clone tissue is not recovered. **(E–H**) Mutant clones of the same genotypes as in **A–D**, except given a survival advantage by making them *Minute* ^+^ in a *Minute* heterozygous background (clones are labelled negatively, by absence of GFP, as in (**A–D**); Hoechst labels nuclei (blue). *rheb* ^—^clones can now be recovered as single cells or small groups of cells (**F**; arrowheads). Double mutant *rheb* ^—^ *wts* clones are slightly larger (**H**), but nowhere near as large as *wild type* (**E**) or *wts* ^—^clones (**G**). **(I–L**) Wings from adult males expressing the following transgenes with *nubbin*.*GAL4*. (**I**) no transgene (*wild type*) (**J**) *UAS*.*ΔP60*, (**K**) *UAS*.*yki*, (**L**) *UAS*.*yki+UAS*.*ΔP60*. Numbers are size ratios compared to *wild type*; in (**L**), bottom italicised number is the size ratio compared to the *ΔP60*-expressing wing in (**J**). (**M–O**) Quantification of wing sizes, cell numbers, and cell sizes from genotypes in (**I–L**). Error bars are Standard Error of the Mean and asterisks denote significances from *t* tests (* = *p* < 0.05, ** = *p* < 0.01, *** = *p* < 0.001, *n*. *s*. = not significant). Number of wings measured = 10 (*wt*), 11 (*ΔP60*), 11 (*Yki*), 11 (*Yki+ΔP60*). Expressing *yki* increased blade size by 34% (**K, M**) via increased cell number (**N**), indicating that Yki accelerates cell growth and cell division rates equally. Expressing a strong inhibitor of the InR pathway component PI3-Kinase (*ΔP60*; [38]) decreased blade size by 66% (**J, M**), due to reductions in both cell size and cell number (**N, O**). When *yki* and *ΔP60* were coexpressed, blade size was still 57% smaller than *wild type* (**L, M**, the slight size increase being manifested in a modest increase in cell number (**N**). The accelerated rates of cell growth and cell division caused by Yki expression are thus equally hindered by *ΔP60* coexpression. **(P–X)** Standard Error of the Mean images of heads composed predominantly of mutant tissue generated using the EGUF/hid technique [68]. (**P–S)** Images are to scale; (**P**) *wild type* (control). (**Q**) *Tor* ^*ΔP*^ causes a “pinhead” phenotype composed of fewer and smaller cells. (**R**) *ex* ^*e1*^ caused substantial overgrowth of the head (note that overgrowth of the eye field does not occur in *ex* mutants [71,72], but the rest of the head capsule is strongly affected). (**S**) *ex* ^*e1*^ *Tor* ^*ΔP*^ again produces a pinhead phenotype, indistinguishable from (**J**). (**T–X**) Images are to scale. (**T**) *akt* ^*q*^, (**U**) *foxo* ^*25*^ *akt* ^*q*^ (**V**) *wts* ^*X1*^, (**W**) *akt* ^*q*^ *wts* ^*X1*^ (**X**) *foxo* ^*25*^ *akt* ^*q*^ *wts* ^*X1*^. InR signalling activates TOR at least in part via the kinase Akt [73], but Akt also controls the phosphorylation and cytoplasmic sequestration of the transcription factor FOXO [74–76]. FOXO is dephosphorylated when InR/Akt activity is diminished and accumulates in the nucleus to suppress cell growth and proliferation [76,77]. Loss of *akt* decreases head size (**T**), an effect which can be partially rescued by simultaneous removal of *foxo* (**U**) [77]. *akt* loss was able to block the *wts* ^*—*^overgrowth phenotype (**V, W**), and importantly, overgrowth was still mostly suppressed when *foxo* was also removed (**X**). These results show that blocking InR pathway activity inhibits Yki-driven cell proliferation largely in a FOXO-independent manner.
(TIF)

S2 Fig Effects of TOR and Rheb overexpression on clone size, cell size, and cell number. **(A–C)** Wing discs from late third instar larvae bearing MARCM clones expressing *UAS*.*p35* (labelled positively with GFP-NLS, green; nuclei are counterstained with Hoechst, blue). The genotypes of clones are (**A**) *UAS*.*p35*, (**B**) *UAS*.*Dp110*+*UAS*.*p35*, (**C**) *UAS*.*Rheb*+*UAS*.*p35*, **D–F**: Quantification of clones sizes, cell numbers, and cell sizes from genotypes in A–C. Error bars are Standard Error of the Mean, and asterisks denote significances from *t* tests (* = *p* < 0.05, ** = *p* < 0.01, *** = *p* < 0.001, *n*. *s*. = not significant). *n* = 50 (*p35*), 34 (*UAS*.*Dp110*+*UAS*.*p35*), 26 (*UAS*.*Rheb*+*UAS*.*p35*). Expression of either *UAS*.*Dp110* or *UAS*.*Rheb* leads to enlarged clone size, through increased cell size and cell number.
(TIF)

S3 Fig TOR and InR pathway inhibition causes nuclear accumulation of Yki. **(A–C)** Wing discs expressing *UAS*.*GFP* in the dorsal compartment with *ap*.*GAL4* labelled with anti-Yki (red), GFP (green) and nuclei (blue; Hoechst). (**A**) Yki is largely cytoplasmic in *wild type* discs, as indicated by modest staining imaged at low magnification. Expressing *Tor* ^*TED*^ (**B**) or *TSC1+2* (**C**) in the dorsal compartment with *ap-GAL4* domain causes some Yki to become nuclear (note gain in staining intensity within the GFP-positive region). (**D–F**) *Tuba1*.*Gal80ts* wing discs expressing *UAS*.*GFP* with *dpp*.*GAL4* for 9 hrs, labelled as in (**A–C**); weak GFP expression results from the short window of GAL4 activity. **(D)** Yki is primarily cytoplasmic in otherwise *wild type* discs. (**E**) Inhibiting InR signalling in GFP cells by coexpressing *UAS-ΔP60* causes some Yki to accumulate in the nucleus. (**F**) Magnification of portion of disc in (**E**) to confirm nuclear accumulation. (**G–J**) Yki localisation (red) is similar in *wild type* **(G)**, *dS6k* ^*—*^ **(H)**, *thor* ^*—*^ **(I)** and *thor* ^—^; *dS6k* ^—^double mutant **(J)** wing discs. Blue in G–J labels Hoechst-stained nuclei.
(TIF)

S4 Fig Effects of Yki knockdown and TOR inhibition on gene expression. **(A, B)** RNAi-mediated knockdown of Yki under *dpp*.*Gal4* control lowers DIAP1 protein (**A**), *diap1* ^*2B2C*^ *-lacZ* (**A’**) and *fj-lacZ* expression (**B**). (**C–G**) Expression of *ex-lacZ* (**C, D**) and Yki (**E–G**) in *ex* ^*e1*^ homozygous mutant wing discs. *UAS*-*GFP* (green) is driven with *dpp-GAL4* (dotted line indicates the A/P boundary) and Hoechst-labelled nuclei are in blue. (**D, F, G**) Expression of *Tor* ^*TED*^ and *TSC1*&*2* under *dpp*.*GAL4* control represses *ex-lacZ* (**D**) while elevating the level of nuclear Yki above that normally seen in *ex* ^*e1*^ homozygous mutant wing discs (**F**), magnified in (**G**). Control discs, expressing only GFP, are shown in **(C, E)**. **(H, I)** Neither *Yki* ^*RNAi*^ nor TOR inhibition repress Vestigial protein in the central blade region, (red: Vg protein). **(J)** TOR inhibition in the Dpp domain has no effect on Armadillo protein (**J**; Arm: magenta), Distal-less protein (**J’**; Dll: red), and a Sd-GFP protein chimaera (**J”**; Sd-GFP: green). (**K)** TOR inhibition has no effect on a *Dll-lacZ* repor*UAS-GFP* was not coexpressed in the discs shown in (**J, K**); dotted line indicates approximate position of the anteroposterior compartment boundary.
(TIF)

S5 Fig Moderate TOR inhibition leads to Yki nuclear accumulation and reduced Yki target gene expression. **(A–C)** Wings from adult females expressing the following transgenes with *nubbin*.*GAL4*: (**A**) *y+* (*wild type*) (**B**) *UAS*.*Rheb* ^*RNAi*^, (**C**) *UAS*.*TOR* ^*RNAi*^. **(D–I)** Moderate TOR inhibition in the Dpp domain by expressing *TOR* ^*TED*^ **(D, E)**, *TOR* ^*RNAi*^ (**F, G**) and *Rheb* ^*RNAi*^ (**H–I**). Dpp domain marked with *UAS*-*GFP* (green and arrowheads) and Hoechst-labelled nuclei are in blue. Moderate to weak nuclear accumulation of Yki is evidenced as gain in Yki staining intensity within the GFP-positive region (**D, F, H**). Expression of the Yki target gene *fj-lacZ* is reduced (**E, G, I**).
(TIF)

S6 Fig Effect of Sd overexpression and Leptomycin B treatment on Yki. **(A–C)** Overexpression of Sd under *dpp*.*Gal4* control causes strong nuclear accumulation of Yki (**A**), and modest repression of the Yki targets DIAP1 (**B**) and *fj-lacZ* (**C**). Yki, DIAP1, and *fj-lacZ* are labelled in red, UAS.GFP expression (green) indicates the region of *dpp*.*Gal4* expression, counterstained with Hoechst. (**D,E**) Cytosolic/nuclear shuttling revealed by incubating *wild type* wing discs in Leptomycin B (LMB) for 1.5 hrs, which inhibits nuclear export: LMB treatment causes increased nuclear accumulation of Yki (red; counterstained with Hoechst).
(TIF)

S7 Fig Parallel metabolic stimulation by TOR is necessary for Yki to drive wing growth. (**A–D**) wing discs from late third instar larvae are shown labelled for Distalless (green), which marks the wing primordium and adjoining portions of the prospective wing hinge, counterstained with Hoechst. (**A**) *wt*, (**B**) *wts* ^*P2*^, (**C**) *ds6k* ^I-I^, (**D**) *ds6k* ^I-I^ *wts* ^*P2*^. Numbers denote disc size ratios compared to *wild type*; in (**D**), the bottom italicised value is a comparison with the *ds6k* genotype. Asterisks denote significances from *t* tests (* = *p* < 0.05, ** = *p* < 0.01, *** = *p* < 0.001, *n*. *s*. = not significant). Number of discs measured = 12 (*wt*), 5 (*wts*), 13 (*ds6k)*, 12 (*ds6k wts*). Yki-driven overgrowth of the wing (caused by removal of *wts*) can be suppressed by removal of S6-Kinase (dS6K; compare discs in (**B**) and (**D**), a TOR target that catalyses cap-dependent mRNA translation, but which is not involved in the nuclear sequestration of Yki (S2H Fig).
(TIF)

S8 Fig Hpo pathway-independent nuclear seclusion of Yki-Sd following TOR inactivation. **(A)** Confocal section of a wing disc taken at the level of nuclei shows Yki protein (red), CD2 (magenta) and Hoechst and Sd-GFP (blue and green respectively) 9 hours following a 1 hr heat shock to excise the Flp-out cassette from an *Act5C>CD2>GAL4* driver (the CD2-expressing cassette has been removed from virtually all cells). GAL4 drives expression of Yki^RNAi^, which strongly depletes Yki protein in clonal tissue (arrows indicate no clonal tissue where normal Yki levels remain). (**B**) ChIP of Yki (black bars) or Sd (blue bars; using an Sd-GFP protein trap chimera in the endogenous Sd locus, and anti-GFP antibody) with mock IP (IgG; green bars) at the *2B2C diap1* enhancer and a control locus (PDH) in *wild type* (*Sd-GFP/ y w hs-flp; Act5C>CD2>GAL4/+*) and *Yki* ^*RNAi*^ discs (*Sd-GFP/ y w hs-flp; UAS-yki* ^*RNAi*^ */+;Act5C>CD2>GAL4*/*+*) following 1 hr heatshock and 8–10 hrs of GAL4 expression. Yki and Sd-GFP are enriched at *2B2C* in *wild type* discs compared to PDH controls. Yki^RNAi^ strongly reduces enrichment of Yki but not Sd-GFP. (**C**) Excising the cassette (magenta) in a *tub>DsRED>Yki-GFP* transgene produces Yki-GFP expression (green), raising total Yki levels 1.69 fold (red), close to the normal physiological range. **(D**) Western blot of phospho-Yki S168 and total Yki in *wild type* control discs (*y w hs-flp; Act5C>CD2>GAL4/+*) and TOR-inhibited (*y w hs-flp; UAS-TorTED/+;Act5C>CD2>GAL4*/*UAS-TSC1&2*) discs following 1 hr heatshock and 8–10 hr of GAL4 expression. 50 discs per lane were dissected; ß-actin is a loading control. (**E, F**) *fj-lacZ* expression (magenta) is still repressed in the Ptc domain (green in rightmost panels of **E** and **F**) by expression *Tor* ^*TED*^ with *ptc-Gal4>* in *wts* ^*X1*^ clones (**E**; marked by absence of GFP in second panel), and *wts* ^*X1*^/*wts* ^*P2*^ homozygous mutant wing discs (**F**). (**G, H**) An Sd-GFP protein trap line (green) shows similar expression in *wild type* discs (**G**) and discs expressing *Tor* ^*TED*^ and *TSC1&2* in the Dpp domain (**H**). Ptc staining approximately marks the Dpp expression domain. (**I–J**) Expressing *TOR* ^*TED*^ and *TSC1+2* in the eye (**I, K**) and leg (**J**) has no clear effect on Yki localisation or Yki target gene expression. The driver *dpp*.*GAL4 UAS-GFP* was used in **I** and **J**, and *Act5C>CD2>GAL4 UAS-GFP* in **K**.
(TIF)

S9 Fig Superphysiological TOR activity does not affect Yki activity but triggers a Yki-independent tumour-suppressive mechanism. (**A, B**) TOR overactivation caused by overexpression of *rheb* with *dpp*.*GAL4* (**A**) or *Act5C>CD2>GAL4* (**B**) (regions marked positively with GFP in green and counterstained with Hoechst in blue) does not influence expression of two different *diap-lacZ* reporters (red). (**C, D**) *Wild type* discs (**C**) and discs experiencing TOR overactivation (**D**; *Tsc1* ^*Q87X*^/*Tsc1* ^*PA23*^) are similar in size, but *Tsc1* ^—^discs show extensive cell death (cleaved caspase III in green; blue: Hoechst). (**F**) *Tsc1* ^*—*^clones (marked by absence of magenta ß-galactosidase) in the eye repress DIAP1 (**F**; red) and *bantam* (**F’**; *bantam* sensor in green). (**G, H**) Overexpressing *bantam* using the EP line *EP(3)3622* with *ptc-GAL4* (**G**; domain labelled by coexpressed ß-galactosidase; magenta), strongly diminishes expression of the *bantam* sensor (**G**’) without noticeable effects on DIAP1 protein levels (**G**; red). Likewise, in **H**, *bantam* homozygous mutant clones (labelled by absence of magenta ß-galactosidase) show a very strong increase in *bantam* sensor levels (green) but no discernable change in DIAP1 protein levels (red). This indicates that native DIAP1 levels are regulated independently of *bantam* in the wing. (**J-N**) *hdc*.*GAL4* discs labelled for cleaved Caspase III (green) and Hoechst (blue). *hdc>rheb* discs (**K**) are the same size as *wild type* (**J**) but show highly elevated levels of cell death. When *diap1* and *rheb* were coexpressed, cell death is fully suppressed (**L**), and disc size increases by 77%; introducing *tub*.GFP-*bantam* while coexpressing *rheb* increases disc size by 70% (**M**), without obviously suppressing cell death. Simultaneously combining *rheb*, *diap1*, and *tub*.GFP-*bantam* together caused the disc to more than double in size (**N**). (**O, P**) Wings from adult female flies of the genotypes: *bantam* ^—^/+ (**O**) and *bantam* ^—^/*bantam* ^—^ *tub*.*GFP-bantam* (**P**). A single copy of *tub*.GFP-*bantam* rescues a *bantam* homozygous mutant animal, producing a normally sized adult.
(TIF)

## Abbreviations

Ex: expanded
FERM: 4.1 Ezrin Radixin Moesin
Hid: head involution defective protein
Hpo: Hippo
ILP: insulin-like peptide
InR: Insulin Receptor
NSF: Nuclear Secluding Factor
Sd: Scalloped
TBD: TEAD binding domain
TEAD: Transcriptional Enhancer Activator Domain
wts: Warts
YAP: YES-Associated Protein
Yki: Yorkie
