# Supplementary figures and images for: Scaling the Drosophila Wing: TOR-Dependent Target Gene Access by the Hippo Pathway Transducer Yorkie
Source: PLoS Biol. 2015 Oct 16;13(10):e1002274. doi: 10.1371/journal.pbio.1002274 (PMC4608745; doi:10.1371/journal.pbio.1002274)

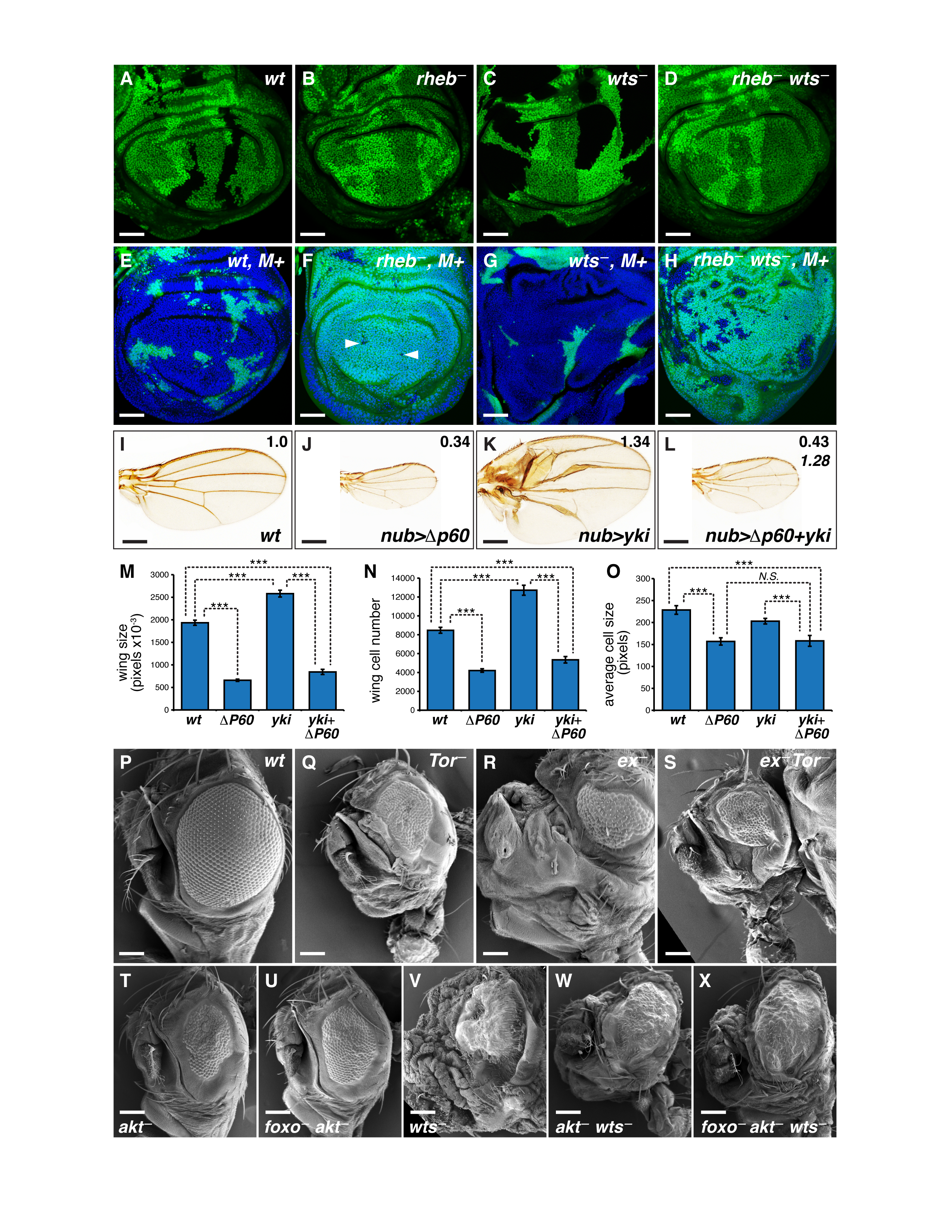

Supplement: S1 Fig — (A–D) Late third instar wing discs with mutant clones marked negatively by absence of GFP (green; wild type sibling clones appear as bright green). (A) wild type (control) (B) rheb AV4, (C) wts X1, (D) rheb AV4 wts X1. Note that in b and d, mutant clone tissue is not recovered. (E–H) Mutant clones of the same genotypes as in A–D, except given a survival advantage by making them Minute + in a Minute heterozygous background (clones are labelled negatively, by absence of GFP, as in (A–D); Hoechst labels nuclei (blue). rheb —clones can now be recovered as single cells or small groups of cells (F; arrowheads). Double mutant rheb — wts clones are slightly larger (H), but nowhere near as large as wild type (E) or wts —clones (G). (I–L) Wings from adult males expressing the following transgenes with nubbin.GAL4. (I) no transgene (wild type) (J) UAS.ΔP60, (K) UAS.yki, (L) UAS.yki+UAS.ΔP60. Numbers are size ratios compared to wild type; in (L), bottom italicised number is the size ratio compared to the ΔP60-expressing wing in (J). (M–O) Quantification of wing sizes, cell numbers, and cell sizes from genotypes in (I–L). Error bars are Standard Error of the Mean and asterisks denote significances from t tests (* = p < 0.05, ** = p < 0.01, *** = p < 0.001, n. s. = not significant). Number of wings measured = 10 (wt), 11 (ΔP60), 11 (Yki), 11 (Yki+ΔP60). Expressing yki increased blade size by 34% (K, M) via increased cell number (N), indicating that Yki accelerates cell growth and cell division rates equally. Expressing a strong inhibitor of the InR pathway component PI3-Kinase (ΔP60; [38]) decreased blade size by 66% (J, M), due to reductions in both cell size and cell number (N, O). When yki and ΔP60 were coexpressed, blade size was still 57% smaller than wild type (L, M, the slight size increase being manifested in a modest increase in cell number (N). The accelerated rates of cell growth and cell division caused by Yki expression are thus equally hindered by ΔP60 coexpression. [file pbio.1002274.s002.tif]

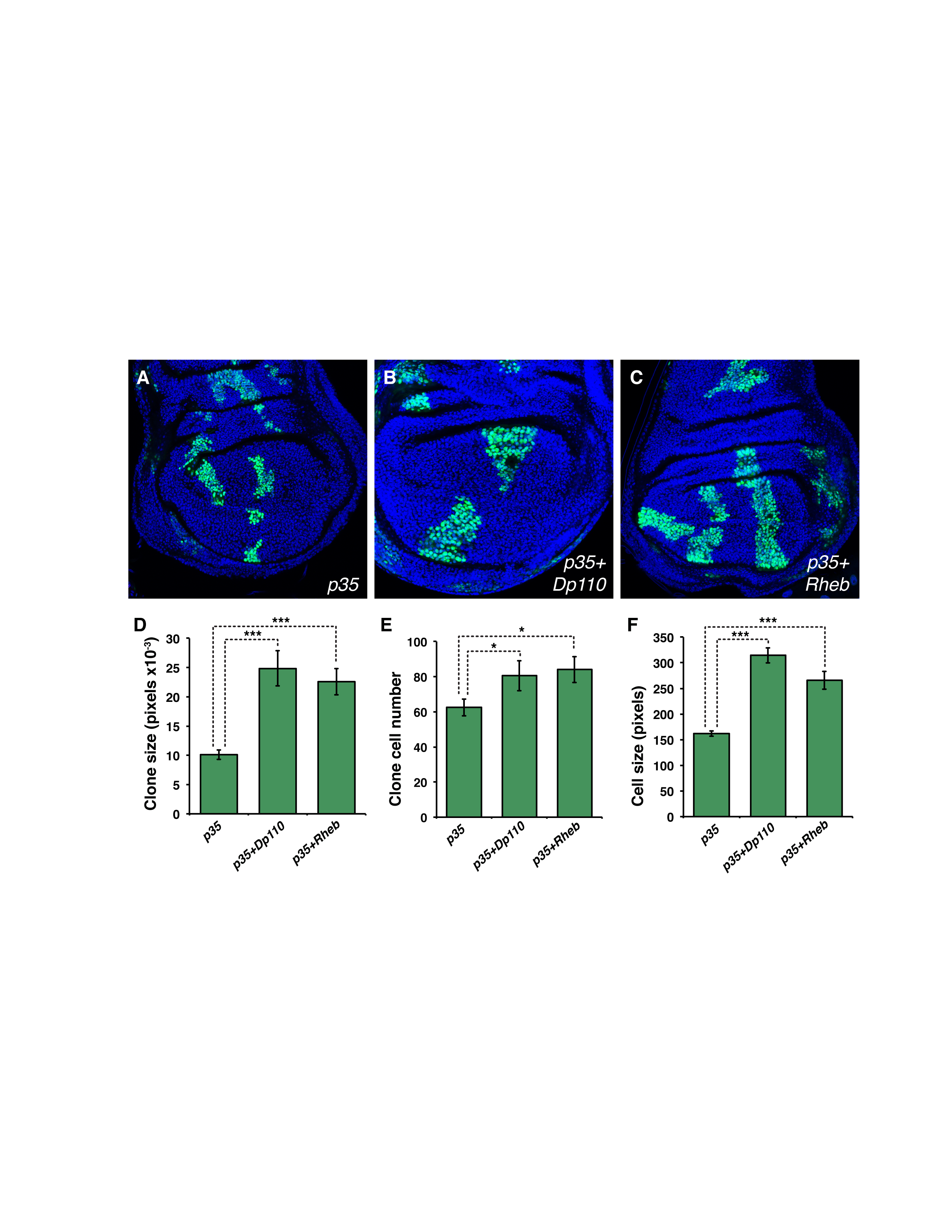

Supplement: S2 Fig — (A–C) Wing discs from late third instar larvae bearing MARCM clones expressing UAS.p35 (labelled positively with GFP-NLS, green; nuclei are counterstained with Hoechst, blue). The genotypes of clones are (A) UAS.p35, (B) UAS.Dp110+UAS.p35, (C) UAS.Rheb+UAS.p35, D–F: Quantification of clones sizes, cell numbers, and cell sizes from genotypes in A–C. Error bars are Standard Error of the Mean, and asterisks denote significances from t tests (* = p < 0.05, ** = p < 0.01, *** = p < 0.001, n. s. = not significant). n = 50 (p35), 34 (UAS.Dp110+UAS.p35), 26 (UAS.Rheb+UAS.p35). Expression of either UAS.Dp110 or UAS.Rheb leads to enlarged clone size, through increased cell size and cell number. (TIF) [file pbio.1002274.s003.tif]

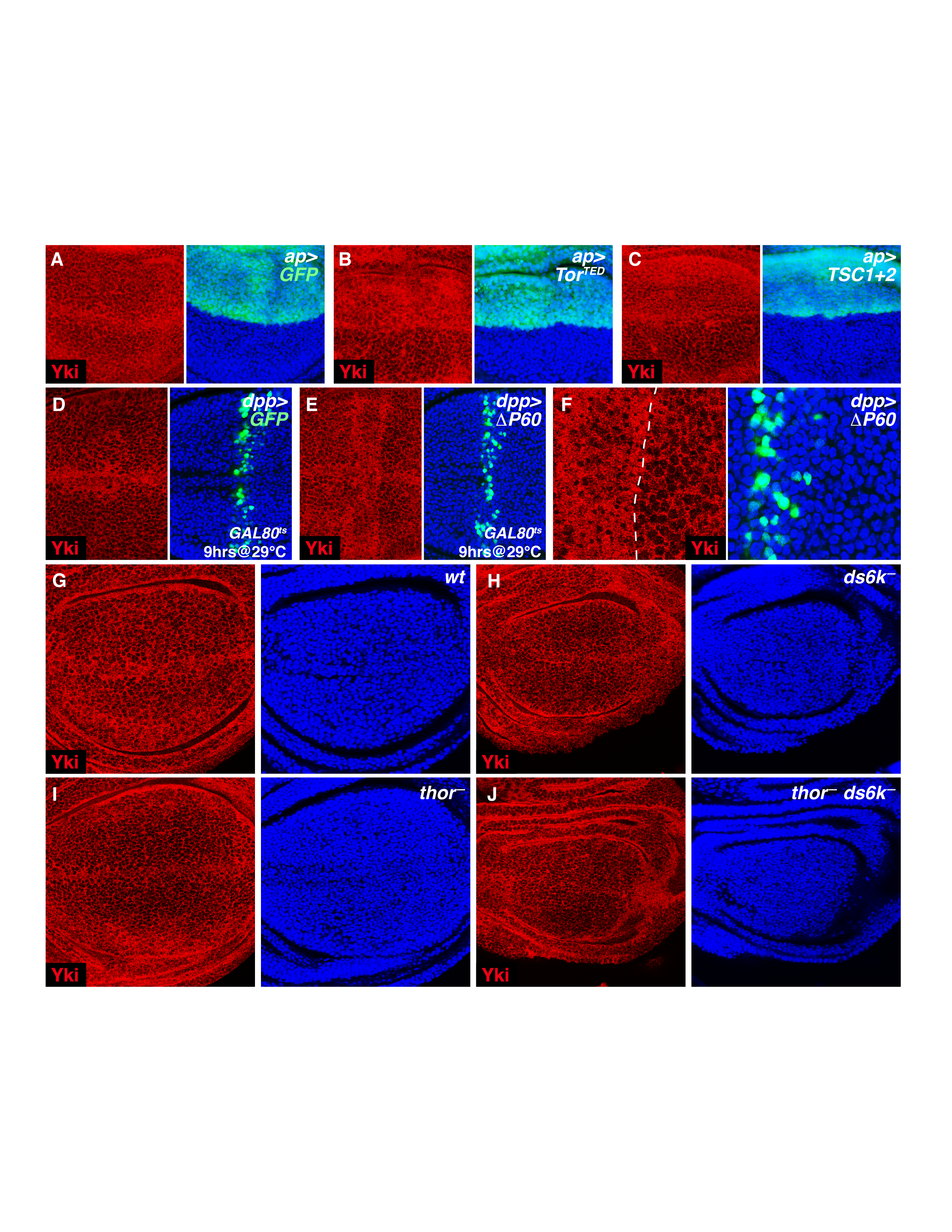

Supplement: S3 Fig — (A–C) Wing discs expressing UAS.GFP in the dorsal compartment with ap.GAL4 labelled with anti-Yki (red), GFP (green) and nuclei (blue; Hoechst). (A) Yki is largely cytoplasmic in wild type discs, as indicated by modest staining imaged at low magnification. Expressing Tor TED (B) or TSC1+2 (C) in the dorsal compartment with ap-GAL4 domain causes some Yki to become nuclear (note gain in staining intensity within the GFP-positive region). (D–F) Tuba1.Gal80ts wing discs expressing UAS.GFP with dpp.GAL4 for 9 hrs, labelled as in (A–C); weak GFP expression results from the short window of GAL4 activity. (D) Yki is primarily cytoplasmic in otherwise wild type discs. (E) Inhibiting InR signalling in GFP cells by coexpressing UAS-ΔP60 causes some Yki to accumulate in the nucleus. (F) Magnification of portion of disc in (E) to confirm nuclear accumulation. (G–J) Yki localisation (red) is similar in wild type (G), dS6k — (H), thor — (I) and thor —; dS6k —double mutant (J) wing discs. Blue in G–J labels Hoechst-stained nuclei. (TIF) [file pbio.1002274.s004.tif]

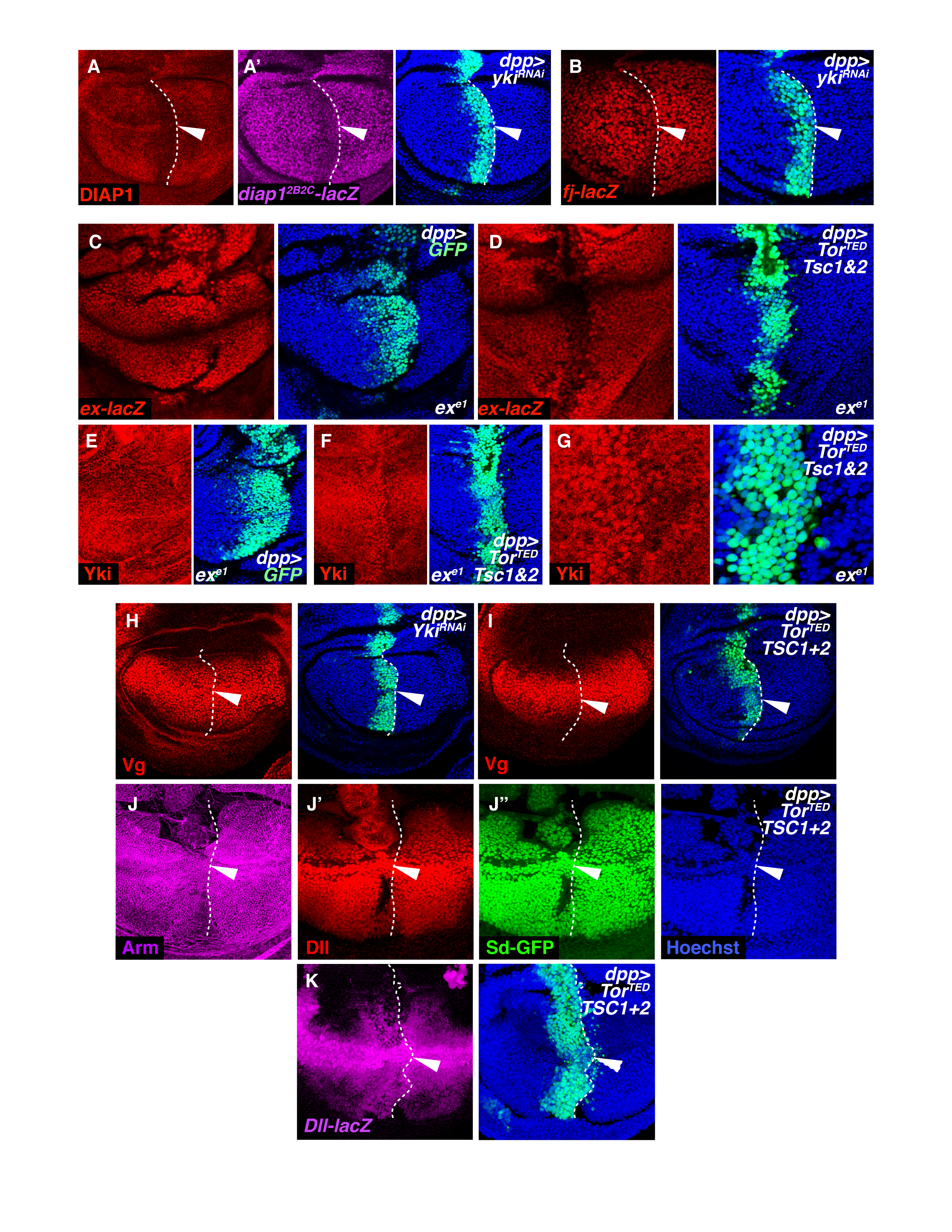

Supplement: S4 Fig — (A, B) RNAi-mediated knockdown of Yki under dpp.Gal4 control lowers DIAP1 protein (A), diap1 2B2C -lacZ (A’) and fj-lacZ expression (B). (C–G) Expression of ex-lacZ (C, D) and Yki (E–G) in ex e1 homozygous mutant wing discs. UAS-GFP (green) is driven with dpp-GAL4 (dotted line indicates the A/P boundary) and Hoechst-labelled nuclei are in blue. (D, F, G) Expression of Tor TED and TSC1&2 under dpp.GAL4 control represses ex-lacZ (D) while elevating the level of nuclear Yki above that normally seen in ex e1 homozygous mutant wing discs (F), magnified in (G). Control discs, expressing only GFP, are shown in (C, E). (H, I) Neither Yki RNAi nor TOR inhibition repress Vestigial protein in the central blade region, (red: Vg protein). (J) TOR inhibition in the Dpp domain has no effect on Armadillo protein (J; Arm: magenta), Distal-less protein (J’; Dll: red), and a Sd-GFP protein chimaera (J”; Sd-GFP: green). (K) TOR inhibition has no effect on a Dll-lacZ reporUAS-GFP was not coexpressed in the discs shown in (J, K); dotted line indicates approximate position of the anteroposterior compartment boundary. (TIF) [file pbio.1002274.s005.tif]

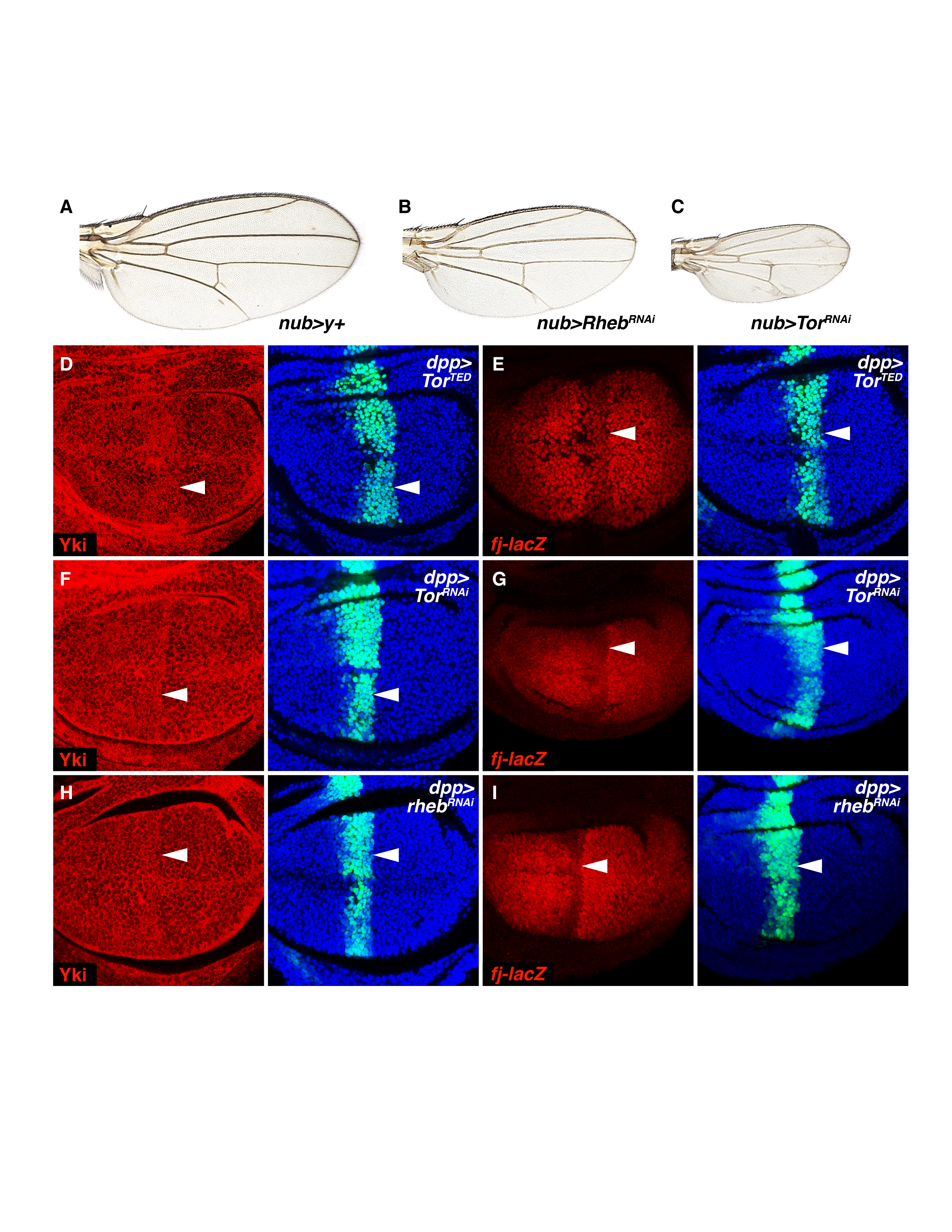

Supplement: S5 Fig — (A–C) Wings from adult females expressing the following transgenes with nubbin.GAL4: (A) y+ (wild type) (B) UAS.Rheb RNAi, (C) UAS.TOR RNAi. (D–I) Moderate TOR inhibition in the Dpp domain by expressing TOR TED (D, E), TOR RNAi (F, G) and Rheb RNAi (H–I). Dpp domain marked with UAS-GFP (green and arrowheads) and Hoechst-labelled nuclei are in blue. Moderate to weak nuclear accumulation of Yki is evidenced as gain in Yki staining intensity within the GFP-positive region (D, F, H). Expression of the Yki target gene fj-lacZ is reduced (E, G, I). (TIF) [file pbio.1002274.s006.tif]

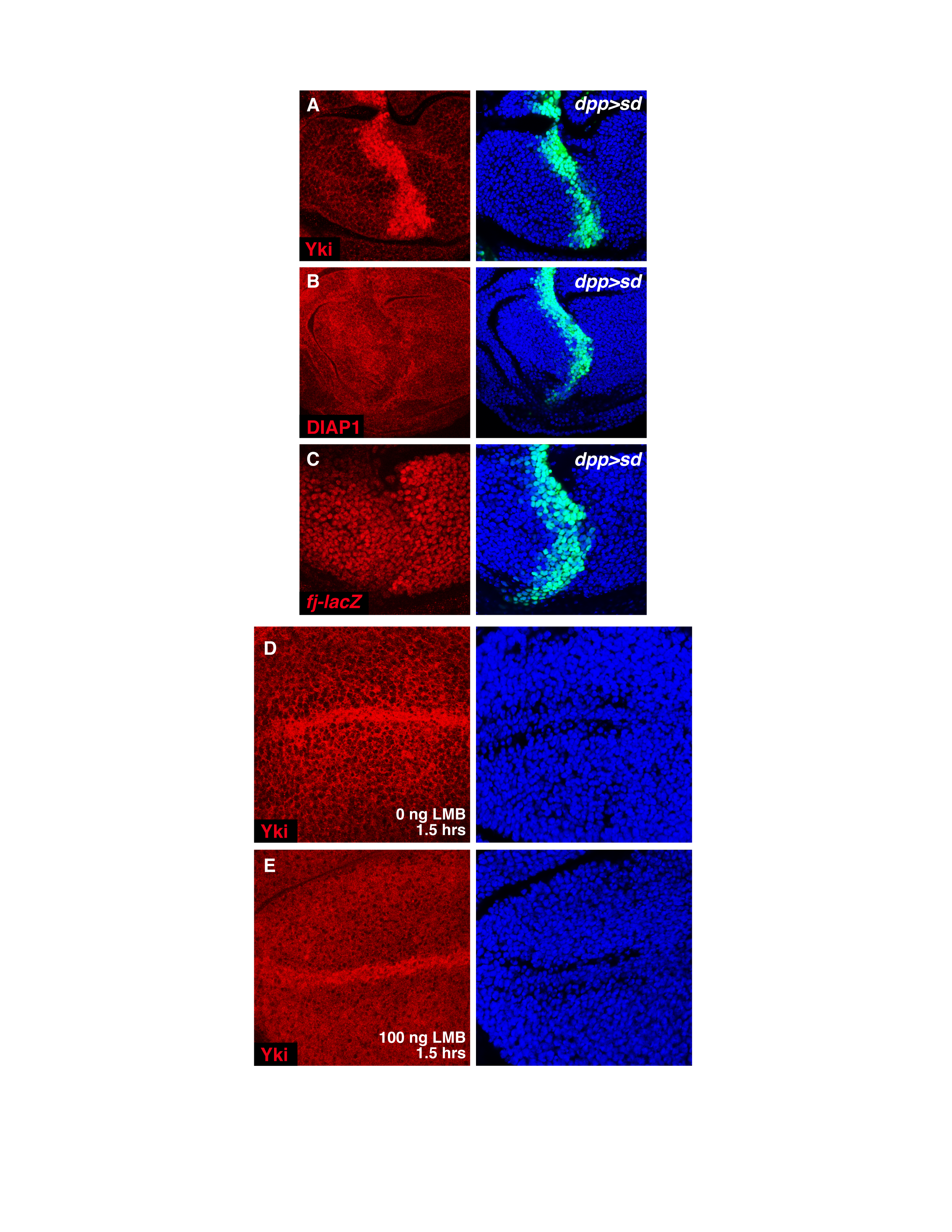

Supplement: S6 Fig — (A–C) Overexpression of Sd under dpp.Gal4 control causes strong nuclear accumulation of Yki (A), and modest repression of the Yki targets DIAP1 (B) and fj-lacZ (C). Yki, DIAP1, and fj-lacZ are labelled in red, UAS.GFP expression (green) indicates the region of dpp.Gal4 expression, counterstained with Hoechst. (D,E) Cytosolic/nuclear shuttling revealed by incubating wild type wing discs in Leptomycin B (LMB) for 1.5 hrs, which inhibits nuclear export: LMB treatment causes increased nuclear accumulation of Yki (red; counterstained with Hoechst). (TIF) [file pbio.1002274.s007.tif]

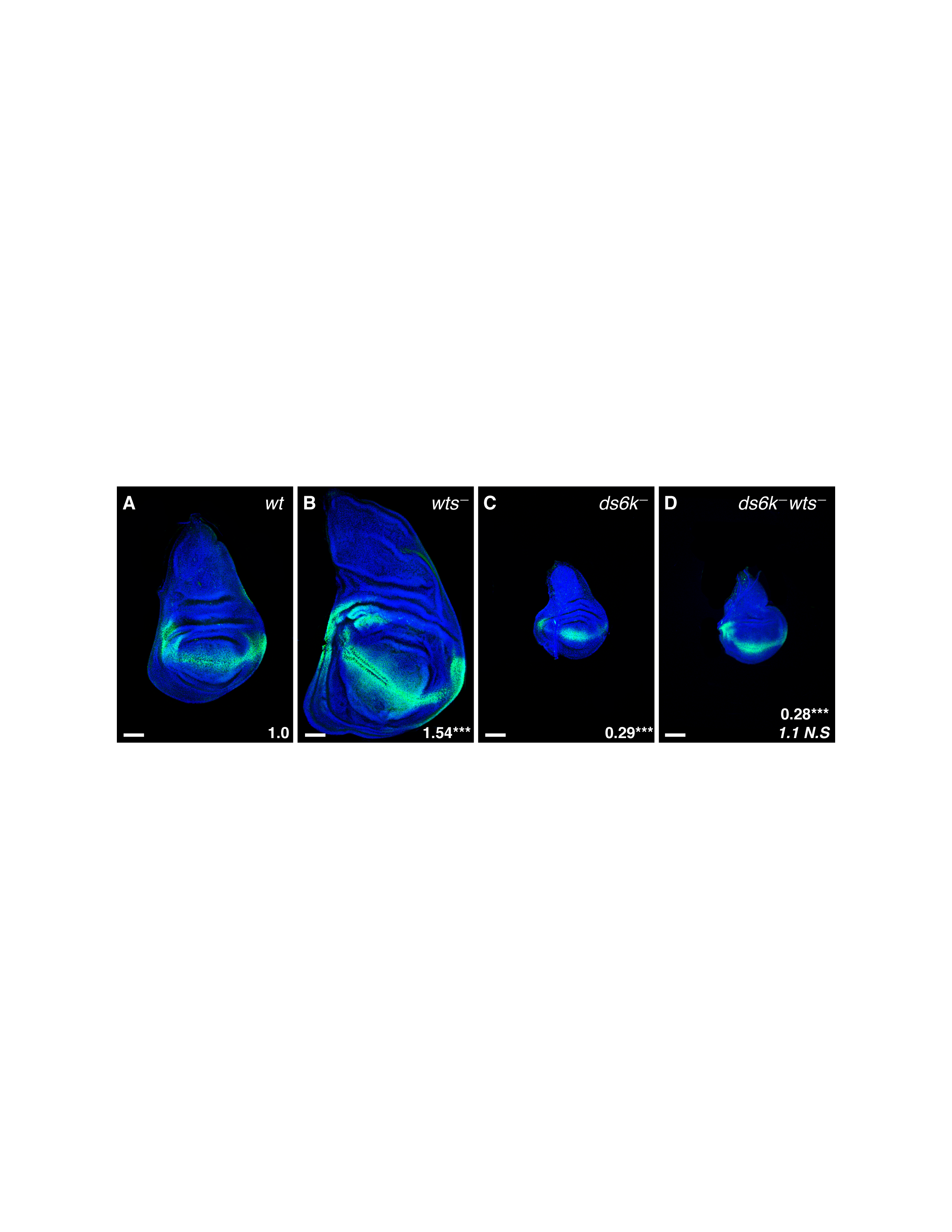

Supplement: S7 Fig — (A–D) wing discs from late third instar larvae are shown labelled for Distalless (green), which marks the wing primordium and adjoining portions of the prospective wing hinge, counterstained with Hoechst. (A) wt, (B) wts P2, (C) ds6k I-I, (D) ds6k I-I wts P2. Numbers denote disc size ratios compared to wild type; in (D), the bottom italicised value is a comparison with the ds6k genotype. Asterisks denote significances from t tests (* = p < 0.05, ** = p < 0.01, *** = p < 0.001, n. s. = not significant). Number of discs measured = 12 (wt), 5 (wts), 13 (ds6k), 12 (ds6k wts). Yki-driven overgrowth of the wing (caused by removal of wts) can be suppressed by removal of S6-Kinase (dS6K; compare discs in (B) and (D), a TOR target that catalyses cap-dependent mRNA translation, but which is not involved in the nuclear sequestration of Yki (S2H Fig). (TIF) [file pbio.1002274.s008.tif]

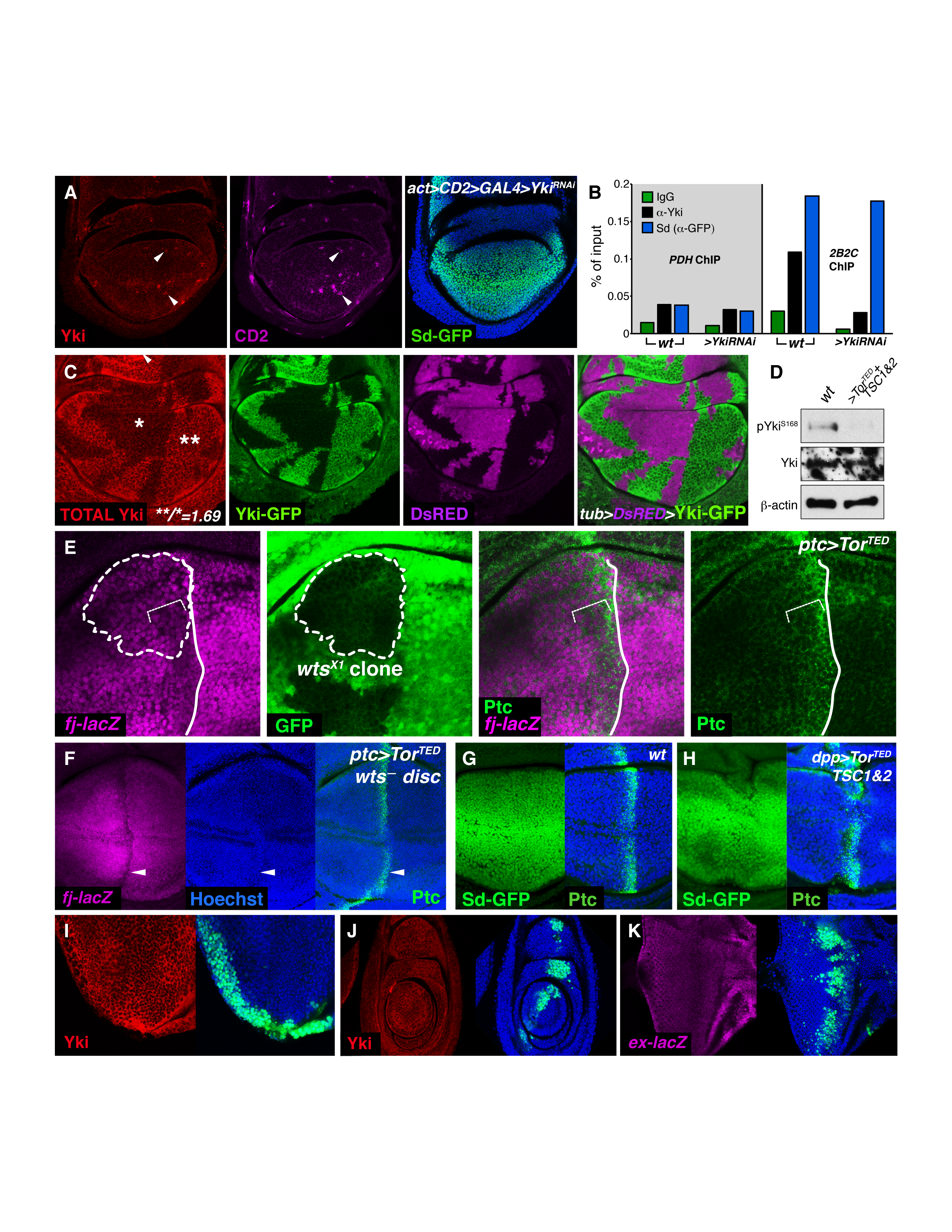

Supplement: S8 Fig — (A) Confocal section of a wing disc taken at the level of nuclei shows Yki protein (red), CD2 (magenta) and Hoechst and Sd-GFP (blue and green respectively) 9 hours following a 1 hr heat shock to excise the Flp-out cassette from an Act5C>CD2>GAL4 driver (the CD2-expressing cassette has been removed from virtually all cells). GAL4 drives expression of YkiRNAi, which strongly depletes Yki protein in clonal tissue (arrows indicate no clonal tissue where normal Yki levels remain). (B) ChIP of Yki (black bars) or Sd (blue bars; using an Sd-GFP protein trap chimera in the endogenous Sd locus, and anti-GFP antibody) with mock IP (IgG; green bars) at the 2B2C diap1 enhancer and a control locus (PDH) in wild type (Sd-GFP/ y w hs-flp; Act5C>CD2>GAL4/+) and Yki RNAi discs (Sd-GFP/ y w hs-flp; UAS-yki RNAi /+;Act5C>CD2>GAL4/+) following 1 hr heatshock and 8–10 hrs of GAL4 expression. Yki and Sd-GFP are enriched at 2B2C in wild type discs compared to PDH controls. YkiRNAi strongly reduces enrichment of Yki but not Sd-GFP. (C) Excising the cassette (magenta) in a tub>DsRED>Yki-GFP transgene produces Yki-GFP expression (green), raising total Yki levels 1.69 fold (red), close to the normal physiological range. (D) Western blot of phospho-Yki S168 and total Yki in wild type control discs (y w hs-flp; Act5C>CD2>GAL4/+) and TOR-inhibited (y w hs-flp; UAS-TorTED/+;Act5C>CD2>GAL4/UAS-TSC1&2) discs following 1 hr heatshock and 8–10 hr of GAL4 expression. 50 discs per lane were dissected; ß-actin is a loading control. (E, F) fj-lacZ expression (magenta) is still repressed in the Ptc domain (green in rightmost panels of E and F) by expression Tor TED with ptc-Gal4> in wts X1 clones (E; marked by absence of GFP in second panel), and wts X1/wts P2 homozygous mutant wing discs (F). (G, H) An Sd-GFP protein trap line (green) shows similar expression in wild type discs (G) and discs expressing Tor TED and TSC1&2 in the Dpp domain (H). Ptc staining approximately marks the Dpp expression domain. [file pbio.1002274.s009.tif]

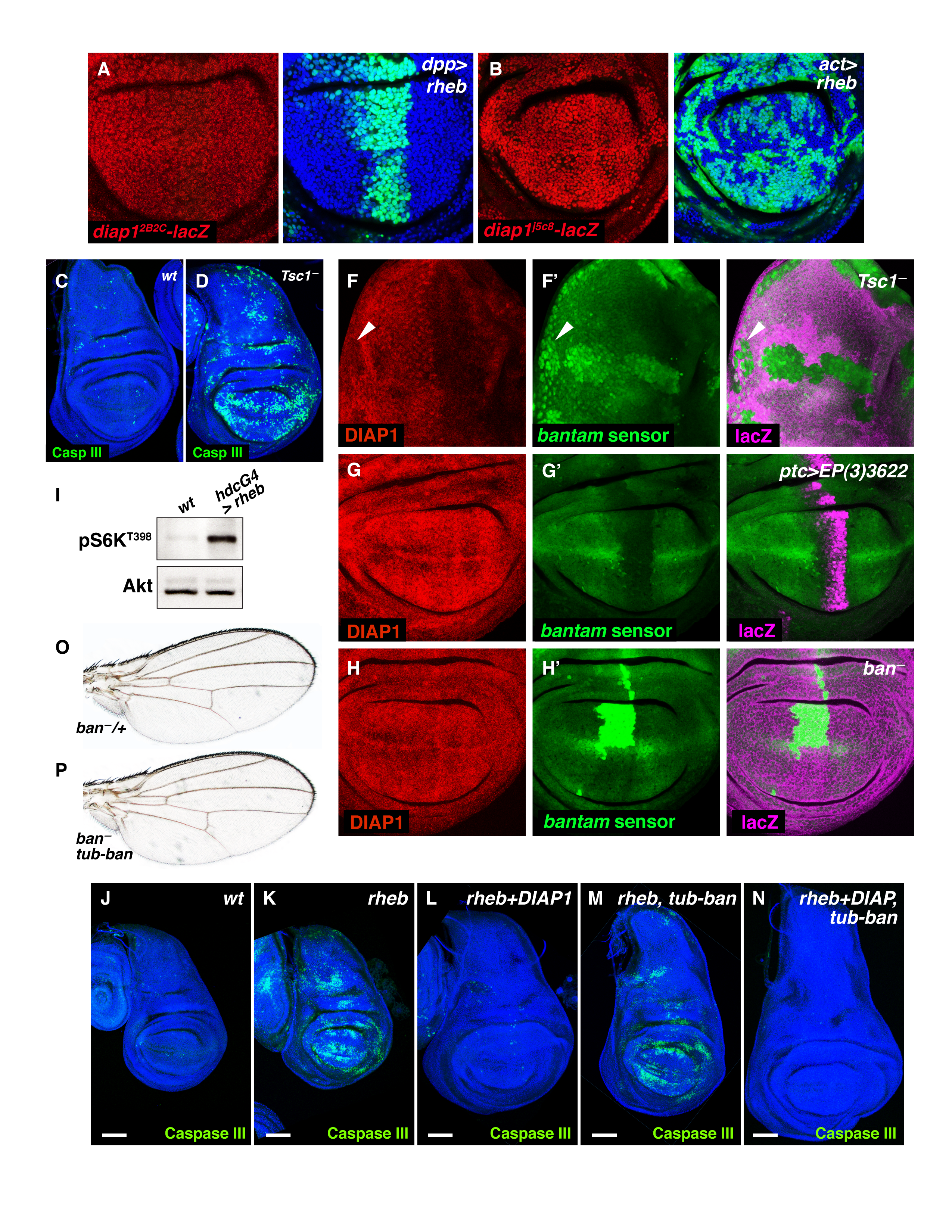

Supplement: S9 Fig — (A, B) TOR overactivation caused by overexpression of rheb with dpp.GAL4 (A) or Act5C>CD2>GAL4 (B) (regions marked positively with GFP in green and counterstained with Hoechst in blue) does not influence expression of two different diap-lacZ reporters (red). (C, D) Wild type discs (C) and discs experiencing TOR overactivation (D; Tsc1 Q87X/Tsc1 PA23) are similar in size, but Tsc1 —discs show extensive cell death (cleaved caspase III in green; blue: Hoechst). (F) Tsc1 —clones (marked by absence of magenta ß-galactosidase) in the eye repress DIAP1 (F; red) and bantam (F’; bantam sensor in green). (G, H) Overexpressing bantam using the EP line EP(3)3622 with ptc-GAL4 (G; domain labelled by coexpressed ß-galactosidase; magenta), strongly diminishes expression of the bantam sensor (G’) without noticeable effects on DIAP1 protein levels (G; red). Likewise, in H, bantam homozygous mutant clones (labelled by absence of magenta ß-galactosidase) show a very strong increase in bantam sensor levels (green) but no discernable change in DIAP1 protein levels (red). This indicates that native DIAP1 levels are regulated independently of bantam in the wing. (J-N) hdc.GAL4 discs labelled for cleaved Caspase III (green) and Hoechst (blue). hdc>rheb discs (K) are the same size as wild type (J) but show highly elevated levels of cell death. When diap1 and rheb were coexpressed, cell death is fully suppressed (L), and disc size increases by 77%; introducing tub.GFP-bantam while coexpressing rheb increases disc size by 70% (M), without obviously suppressing cell death. Simultaneously combining rheb, diap1, and tub.GFP-bantam together caused the disc to more than double in size (N). (O, P) Wings from adult female flies of the genotypes: bantam —/+ (O) and bantam —/bantam — tub.GFP-bantam (P). A single copy of tub.GFP-bantam rescues a bantam homozygous mutant animal, producing a normally sized adult. (TIF) [file pbio.1002274.s010.tif]
